# Evaluating CD133 as a Radiotheranostic Target in Small-Cell Lung Cancer

**DOI:** 10.1021/acs.molpharmaceut.3c01063

**Published:** 2024-02-08

**Authors:** Samantha M. Sarrett, Cindy Rodriguez, Samantha Delaney, Meena M. Hosny, Joni Sebastiano, Ana Santos-Coquillat, Outi M. Keinänen, Lukas M. Carter, Kristin J. Lastwika, Paul D. Lampe, Brian M. Zeglis

**Affiliations:** † Department of Chemistry, Hunter College, City University of New York, New York, New York 10065, United States; ‡ Ph.D. Program in Biochemistry, The Graduate Center of the City University of New York, New York, New York 10016, United States; § Department of Radiology, Memorial Sloan Kettering Cancer Center, New York, New York 10065, United States; ∥ Ph.D. Program in Chemistry, The Graduate Center of the City University of New York, New York, New York 10016, United States; ⊥ Department of Chemistry, CICECO—Aveiro Institute of Materials, University of Aveiro, Campus Universitario de Santiago, Aveiro 3810-193, Portugal; # Department of Chemistry, University of Helsinki, Helsinki 00100, Finland; ¶ Translational Research Program, Public Health Sciences Division, Fred Hutchinson Cancer Research Center, Seattle, Washington 98109, United States; ∇ Translational Science and Therapeutics Division, Fred Hutchinson Cancer Research Center, Seattle, Washington 98109, United States; ○ Human Biology Division, Fred Hutchinson Cancer Research Center, Seattle, Washington 98109, United States

**Keywords:** PET imaging, radioimmunotherapy, orthotopic xenograft, metastatic xenograft, patient-derived xenograft, CD133, small-cell lung cancer

## Abstract

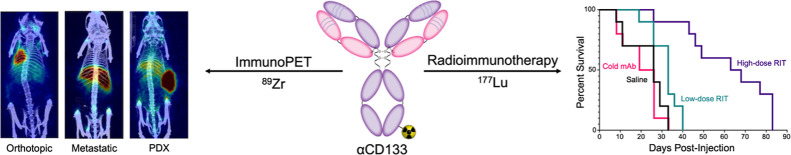

Despite decades of work, small-cell lung cancer (SCLC) remains a frustratingly recalcitrant disease. Both diagnosis and treatment are challenges: low-dose computed tomography (the approved method used for lung cancer screening) is unable to reliably detect early SCLC, and the malignancy’s 5 year survival rate stands at a paltry 7%. Clearly, the development of novel diagnostic and therapeutic tools for SCLC is an urgent, unmet need. CD133 is a transmembrane protein that is expressed at low levels in normal tissue but is overexpressed by a variety of tumors, including SCLC. We previously explored CD133 as a biomarker for a novel autoantibody-to-immunopositron emission tomography (PET) strategy for the diagnosis of SCLC, work that first suggested the promise of the antigen as a radiotheranostic target in the disease. Herein, we report the *in vivo* validation of a pair of CD133-targeted radioimmunoconjugates for the PET imaging and radioimmunotherapy of SCLC. To this end, [^89^Zr]Zr-DFO-αCD133 was first interrogated in a trio of advanced murine models of SCLC—i.e., orthotopic, metastatic, and patient-derived xenografts—with the PET probe consistently producing high activity concentrations (>%ID/g) in tumor lesions combined with low uptake in healthy tissues. Subsequently, a variant of αCD133 labeled with the β-emitting radiometal ^177^Lu—[^177^Lu]Lu-DTPA-A″-CHX-αCD133—was synthesized and evaluated in a longitudinal therapy study in a subcutaneous xenograft model of SCLC, ultimately revealing that treatment with a dose of 9.6 MBq of the radioimmunoconjugate produced a significant increase in median survival compared to a control cohort. Taken together, these data establish CD133 as a viable target for the nuclear imaging and radiopharmaceutical therapy of SCLC.

## Introduction

Lung cancer is the second most frequently diagnosed malignancy in the world, constituting ∼12% of annual global cancer diagnoses.^1−3^ Small-cell lung cancer (SCLC) accounts for nearly 13% of lung cancers and has a dismal 5 year survival rate of ∼7%. Those diagnosed at the early (or limited) stage of the disease have a 5 year survival rate of nearly ∼30%; however, low-dose computed tomography (CT)—the current gold standard for detecting lung cancer—cannot reliably detect SCLC at this critical early stage.^4^ Furthermore, while [^18^F]FDG-based positron emission tomography (PET) has been used to stage SCLC, it has also performed poorly at screening for early disease.^5^ In light of these shortcomings, close to 70% of patients present with metastatic lesions at the time of diagnosis.^6^ The current standard of care for SCLC is predicated on chemotherapy, chemoradiation, or chemotherapy coupled with immunotherapy.^7^ However, despite these treatments, most patients relapse and require second-line chemotherapy, after which most succumb to the disease. Clearly, there is a critical need for new diagnostic and therapeutic tools for the management of SCLC.

CD133—also known as prominin-1—is an integral membrane protein composed of five transmembrane regions, two glycosylated extracellular loops, and two cysteine-rich intracellular loops that is typically found in cholesterol-rich protrusions on the surface of cells.^8,9^ CD133 was originally discovered in the microvilli of neuroepithelial and hematopoietic stem cells, paving the way for its use as a stem cell biomarker.^10,11^ Indeed, several clinical studies have focused on using CD133+ stem cells for therapy for liver cirrhosis, myocardial repair, and spinal cord injury repair.^12−15^ CD133 is also overexpressed in a wide variety of malignancies, including colon, kidney, liver, lung, ovary, prostate, pancreas, and skin cancers.^16^ Critically, healthy tissues express far lower levels of protein, making it a promising target for both imaging and therapy. We previously explored the potential of CD133 as a biomarker for the early diagnosis and molecular imaging of patients with SCLC.^17^ We found that CD133 is significantly overexpressed in patients with SCLC and that the expression rate of the protein does not vary with the stage of the disease. Furthermore, we determined that CD133-targeting autoantibodies could be observed in the plasma of patients up to one year prior to their diagnosis, underscoring the viability of the protein as an early marker of SCLC. Finally, we synthesized a CD133-targeting radioimmunoconjugate labeled with the positron-emitting radiometal zirconium-89 (*t*_1/2_ ∼ 3.3 day)—[^89^Zr]Zr-DFO-αCD133—and performed pilot immunoPET experiments in a subcutaneous xenograft model of SCLC that demonstrated the selective uptake of the probe in tumor tissue. We concluded that CD133 could lie at the heart of an autoantibody-to-immunoPET paradigm for the early diagnosis and visualization of the disease.^17^

Herein, we describe the systematic exploration of CD133 as a target for the nuclear imaging and radiopharmaceutical therapy of SCLC. Given that our pilot data were collected in a somewhat simplistic subcutaneous xenograft model of SCLC, we began by evaluating the pharmacokinetic profile of [^89^Zr]Zr-DFO-αCD133 in a trio of murine models that better recapitulate human disease: orthotopic, metastatic, and patient-derived xenografts (PDXs). Subsequently, we synthesized and validated a variant of αCD133 labeled with the β-particle emitting radiometal lutetium-177 (^177^Lu; *t*_1/2_ ∼6.7 days)—[^177^Lu]Lu-DTPA-A″-CHX-αCD133—and interrogated its efficacy as a radioimmunotherapeutic in a subcutaneous xenograft model of SCLC. Ultimately, the data suggest that CD133 is a promising radiotheranostic target in SCLC that may merit further examination in the clinic.

## Methods and Materials

### General

All reagents were purchased from Fisher Scientific (Thermo Fisher Scientific; Waltham, MA, USA) unless otherwise noted. αCD133 was provided by the Paul Lampe Laboratory at the Fred Hutchinson Cancer Center. Protein concentrations were determined via UV–vis spectroscopy using a molar absorptivity at 280 nm of 2.1 × 10^5^ M^–1^ cm^–1^ and a molecular weight of 1.5 × 10^5^. All the water used was ultrapure (>18.2 MΩ·cm at 25 °C). *p*-SCN-Bn-DFO and *p*-SCN-Bn-CHX-A″-DTPA were purchased from Macrocyclics, Inc. (Plano, TX, USA). MALDI mass spectrometry was performed by the Alberta Proteomics and Mass Spectrometry Facility (University of Alberta; Edmonton, AB, Canada). ^89^Zr was provided by 3D Imaging (Little Rock, AR, USA), and ^177^Lu was provided by ITM Radiopharma (Munich, Germany).

### Instrumentation

All instruments were calibrated and maintained according to the standard quality control practices and procedures. UV–vis measurements were taken on a Shimadzu BioSpec-nano microvolume UV–vis spectrophotometer (Shimadzu Scientific Instruments; Kyoto, Japan). Radioactivity measurements were taken using a CRC-15R dose calibrator (Capintec, Inc.; Ramsey, NJ, USA) and an automatic Wizard^2^gamma counter (PerkinElmer; Waltham, MA, USA). Surface plasmon resonance was performed using a Nicoya OpenSPR-XT instrument (Nicoya Lifesciences; Kitchener, ON, Canada).

### Synthesis of DFO-αCD133

DFO-αCD133 was prepared as reported previously.^17,18^ In brief, αCD133 (1.0 mg) in Chelex 100-treated (Bio-Rad Laboratories; Hercules, CA, USA) phosphate-buffered saline (Chelex-PBS, pH 7.4) was diluted to a final concentration of 1.0 mg/mL. The pH of the solution was adjusted to 8.8–9.0 with 0.1 M Na_2_CO_3_, 35 equiv of *p*-SCN-Bn-DFO (7.05 μL, 25 mg/mL in DMSO) were added in small aliquots, and the resulting solution was incubated on a ThermoMixer (37 °C, 500 rpm, 1 h). The immunoconjugate was purified using size exclusion chromatography (PD-10 column; GE Healthcare; Chicago, IL, USA), eluted with 2 mL of Chelex-PBS, pH 7.4, and concentrated using 2 mL Amicon Ultra centrifugal filters with a 50 kDa molecular weight cutoff (MWCO; MilliporeSigma).

### Synthesis of ^degly^DFO-αCD133

DFO-αCD133 was deglycosylated for all of the studies involving NSG mice. Deglycosylation was performed using Remove-iT PNGase F (New England Biolabs) according to the manufacturer’s instructions. In brief, newly synthesized DFO-αCD133 (1.0 mg, 4 mg/mL, 200 μL) in Chelex-PBS, pH 7.4, was added to an Eppendorf tube with 40 μL of 10 × G7 reaction buffer and 7 μL of PNGase F (1.5 enzyme: monoclonal antibody (mAb) ratio). The solution was then diluted to 400 μL with deionized (DI) water and incubated on a ThermoMixer at 37 °C and 400 rpm overnight. The next morning, the reaction mixture was purified by using chitin beads. To this end, ∼50 μL of chitin beads was first added to a microcentrifuge tube and washed 5× with 500 μL of PBS using a magnetic rack to settle the beads after each wash. The mAb reaction mixture was then added to the beads, and the solution was mixed thoroughly and incubated on ice for 15 min. Following this incubation, a magnetic rack was used to settle the beads, and the supernatant was collected into an Amicon centrifugal filter with a 50 kDa MWCO. The beads were then washed 3×, and the supernatant was added to the centrifugal filter containing the reaction mixture after each wash. Finally, the purified mAb was concentrated by using a centrifugal filter.

### Synthesis of DTPA-A″-CHX-αCD133

DTPA-A″-CHX-αCD133 was prepared in a manner similar to DFO-αCD133. In brief, αCD133 (1.0 mg) in Chelex-PBS, pH 7.4, was diluted to a final concentration of 1.0 mg/mL. The pH of the solution was adjusted to 8.8–9.0 with 0.1 M Na_2_CO_3_, 50 equiv of *p*-SCN-Bn-CHX-A″-DTPA (9.90 μL, 25 mg/mL in DMSO) were added in small aliquots, and the resulting solution was incubated on a ThermoMixer (37 °C, 500 rpm, 1 h). The immunoconjugate was purified using size exclusion chromatography, eluted with 2 mL of Chelex-PBS at pH 7.4, and concentrated using a 2 mL Amicon Ultra centrifugal filter with a 50 kDa MWCO (MilliporeSigma).

### Radiolabeling with Zirconium-89

DFO-αCD133 or ^degly^DFO-αCD133 was radiolabeled with [^89^Zr]Zr^4+^ according to the standard published protocols.^18^ In brief, each immunoconjugate (0.5 mg) was diluted in Chelex-treated PBS to a final concentration of 0.5 mg/mL. [^89^Zr]Zr^4+^ [92.5–370 MBq (2.5–10 mCi)] in 1.0 M oxalic acid was then diluted with Chelex-treated PBS, and the solution pH was adjusted to 7.0–7.5 with 1.0 M Na_2_CO_3_ (final volume: 100 μL). After the bubbling of CO_2_ stopped, the ^89^Zr solution was added to the antibody solution, mixed thoroughly, and incubated on a ThermoMixer for 15 min at 500 rpm and 37 °C. The progress of the reaction was monitored via radio-instant thin layer chromatography (iTLC) with an eluent of 50 mM ethylenediaminetetraacetic acid (EDTA), pH 5.0, an AR-2000 Radio-TLC plate reader, and Winscan Radio-TLC software (Bioscan, Inc.; Washington, DC, USA). Once the reaction reached completion, free [^89^Zr]Zr^4+^ was removed via size exclusion chromatography. The radiochemical purity of the final radiolabeled construct was assayed using radio-iTLC with an eluent of 50 mM EDTA, pH 5.0.

### Radiolabeling with Lutetium-177

DTPA-A″-CHX-αCD133 in Chelex-PBS, pH 7.4 (0.5 mg, 2.5 mg/mL, 200 μL), was diluted with 800 μL of ammonium acetate (0.25 M, pH 5.5) to a final concentration of 0.5 mg/mL in 1 mL. Next, [^177^Lu]LuCl_3_ in 0.05 M HCl [185 MBq (5 mCi)] was added to the mAb solution, and the reaction mixture was incubated on a ThermoMixer at 37 °C and 500 rpm for 1 h. The progress of the reaction was monitored via radio-iTLC with an eluent of 50 mM EDTA, pH 5.0, an AR-2000 Radio-TLC plate reader, and Winscan Radio-TLC software. Once the reaction reached completion, free [^177^Lu]Lu^3+^ was removed via size exclusion chromatography. The radiochemical purity of the final radiolabeled construct was assayed using radio-iTLC with an eluent of 50 mM EDTA, pH 5.0.

### Cell Culture

The human SCLC cell line NCI–H82 (“H82”) was purchased from the American Type Culture Collection in 2020 and periodically authenticated by the Specimen Processing/Research Cell Bank Shared Resource using the short tandem repeat combined DNA Index System typing. Cells were maintained in Dulbecco’s modified Eagle’s medium supplemented with 10% heat-inactivated fetal calf serum, 100 units/mL penicillin, 100 units/mL streptomycin, 2 mM l-glutamine, 10 mM HEPES, 4.5 g/L d-glucose, 1.5 g/L sodium bicarbonate, and 1 mM sodium pyruvate in an incubator at 37 °C and 5% CO_2(g)_. Cells were passaged upon reaching 80% confluency. Aggregates that may have formed in suspension were dissociated by incubating the cells with Gibco TrypLE Express enzyme (1×) with phenol red (ThermoFisher) for 5 min between each passage. The cells were also strained using cell strainers (MACS SmartStrainers, 30 and 70 μm; Miltenyi Biotec; Auburn, CA, USA) to ensure a homogeneous cell suspension mixture prior to any experiments or xenografting.

### Production and Culture of H82-*luc* Cells

H82 cells were directly transfected with 2 μg of pcDNA3.1(+)/Luc2 = tdT plasmid (Addgene #32904) using Nucleofector Kit L (Lonza VVCA-1005) and program A-020. Transfected cells were selected for 7 days using 1000 μg/mL G418 (Invitrogen) and subsequently sorted using fluorescence-activated cell sorting for tomato red fluorescent protein-positive cells to >95% purity. The H82-*luc* cells were cultured and maintained, as described above.

### Animal Care

Five to eight-week-old female athymic nude mice (Jackson Laboratory #007850) or NSG mice (Jackson Laboratory #005557) were allowed to acclimatize approximately 1 week prior to inoculation. Animals were housed in ventilated cages and given food and water ad libitum. All animal work was approved by the Institutional Animal Care and Use Committees (IACUCs) of Hunter College and Weill Cornell Medical College.

### Subcutaneous Xenografts

Subcutaneous xenografts were used for the longitudinal radioimmunotherapy study. Athymic nude mice were anesthetized by inhalation of a 2% isoflurane/oxygen gas mixture (Baxter Healthcare; Deerfield, IL, USA). The injection site was sanitized with an ethanol wipe, and 3 × 10^6^ H82-*luc* cells (150–200 μL) in media with 1:1 Matrigel (Corning Life Sciences; Corning, NY, USA) were injected subcutaneously in the right flank. The H82-*luc* tumors reached an acceptable size for experimentation (∼100 mm^3^) after approximately 2 weeks.

### Orthotopic Xenografts

Athymic nude mice were anesthetized by inhalation of a 2% isoflurane/oxygen gas mixture (Baxter Healthcare; Deerfield, IL, USA). The implantation of the cells into the lungs was performed by the Memorial Sloan Kettering Antitumor Assessment Core under IACUC-approved protocols. In brief, an incision was made under the left scapula, and 1 × 10^6^ H82-*luc* cells (40 μL) were injected into the parenchyma of the left lung. To ensure homogeneous tumors, the cell suspension was mixed thoroughly prior to each inoculation. The growth of the H82-*luc* tumors was monitored via bioluminescence imaging (BLI), and they reached an acceptable size for experimentation (i.e., tumor signal >1 × 10^6^ p/s/cm^2^/sr) after approximately 4 weeks.

### Metastatic Xenografts

NSG mice were placed into a mouse restrainer and warmed with a heat lamp in order to promote dilation of the lateral tail vein; 1 × 10^6^ H82-*luc* cells (100 μL in saline) were injected into the lateral tail vein of the mice. The growth of the metastatic H82-*luc* lesions was monitored via BLI, and they reached an acceptable size for experimentation (i.e., tumor signal >1 × 10^6^ p/s/cm^2^/sr) after approximately 18 days.

### Patient-Derived Xenografts

PDX samples were provided by the laboratory of Charles Rudin at the Memorial Sloan Kettering Cancer Center (MSKCC). The tumors were implanted into the right flank of nude athymic mice by the Memorial Sloan Kettering Antitumor Assessment Core under IACUC-approved protocols.

### PET Imaging

PET images of the mice bearing subcutaneous xenografts were acquired by using a microPET Focus 120 (Siemens Medical Solutions). Nude mice (*n* = 4) underwent static scans between 24 and 144 h after the intravenous tail vein administration of [^89^Zr]Zr-DFO-αCD133 [3.7–3.9 MBq (100–105 μCi), 10–10.5 μg in 100 μL of PBS] for a total scan time of 10–30 min. The counting rates in the reconstructed images were converted to activity concentrations (percentage injected dose per gram of tissue [%ID/g]) by using a system calibration factor derived from the imaging of a mouse-sized water-equivalent phantom containing ^89^Zr. Image reconstruction was performed via 3-dimensional ordered subset expectation maximization (3D-OSEM). The resulting images were processed using ASIPro VMTM software (Concorde Microsystems). Imaging with a nonspecific, isotype control radioimmunoconjugate—[^89^Zr]Zr-DFO-antihuman immunoglobulin G antibody—was previously performed in a subcutaneous model of SCLC to demonstrate the specificity of [^89^Zr]Zr-DFO-αCD133, abrogating the need for such experiments in this investigation.^17^

PET images of the mice bearing orthotopic xenografts were obtained using an Inveon PET/CT small animal imaging system (Siemens Medical Solutions; Malvern, PA, USA). Nude mice (*n* = 4) underwent static scans between 24 and 144 h after the intravenous tail vein administration of [^89^Zr]Zr-DFO-αCD133 [3.7–3.9 MBq (100–105 μCi), 5–5.5 μg in 100 μL of PBS] for a total scan time of 10–30 min. The images were reconstructed, processed as described above, and analyzed using Inveon Research Workplace software.

PET images of the mice bearing metastatic and PDXs were obtained using an Inveon PET/CT small animal imaging system. NSG mice (*n* = 4) underwent static scans between 24 and 168 h after the intravenous tail vein administration of [^89^Zr]Zr-^degly^DFO-αCD133 [3.7–3.9 MBq (100–105 μCi), 5–5.5 μg in 100 μL of PBS] for a total scan time of 10–30 min. The images were reconstructed, processed as described above, and analyzed using Inveon Research Workplace software.

### Biodistribution Studies

Following the final time point of the PET imaging studies (144 h for the orthotopic and subcutaneous models; 168 h for the metastatic and PDX models), the mice were euthanized via CO_2(g)_ asphyxiation, followed by cervical dislocation. Selected organs were collected, rinsed in water, dried, weighed, and quantified using an ^89^Zr-calibrated automatic Wizard^2^γ-counter (PerkinElmer). For certain tumor models, the lungs and livers were handled differently for tissue slide preparation (see Supporting Information Methods for details). The counts per minute in each tissue were corrected for background and decay to the start of the activity measurement. The %ID/g for each sample was calculated through normalization to the total injected activity.

### Longitudinal Radioimmunotherapy Study

Mice bearing subcutaneous ∼100 mm^3^ H82-*luc* tumors were randomly sorted into 5 cohorts. After randomization, control cohorts were injected via the lateral tail vein with either 0.9% sterile saline (100 μL, *n* = 10) or unlabeled DTPA-A″-CHX-αCD133 (50 μg in 100 μL of PBS, *n* = 10). The treatment cohorts were administered activities of [^177^Lu]Lu-DTPA-A″-CHX-αCD133 (*n* = 10 per dose) of either 4.6 MBq (125 μCi, 100 μL in PBS, 50 μg) or 9.3 MBq (250 μCi, 100 μL in PBS, 50 μg). The activities for administration were selected based on murine dosimetry estimates obtained from [^89^Zr]Zr-DFO-αCD133 PET images (Table S4) and accepted absorbed dose thresholds for tumor response and normal tissue tolerance.^19−21^ Following injection, the weight of the mice and the dimensions of the subcutaneous tumors were measured twice a week. Once a week—starting the week before the injection of the radioimmunoconjugate—50 μL of blood was collected from representative mice (*n* = 3) in each cohort via the lateral tail vein and subsequently analyzed using a Hemavet (Element HT5, Heska). The IACUC-approved end points for the study were (i) if a mouse were to lose or gain >10% of body weight between measurements, (ii) if a mouse’s tumor were to grow to a volume of >2000 mm^3^ between measurements, (iii) or if a mouse were to become lethargic and/or skeletal in appearance.

## Results and Discussion

### Synthesis and Characterization of [^89^Zr]Zr-DFO-αCD133

**Figure 1:**
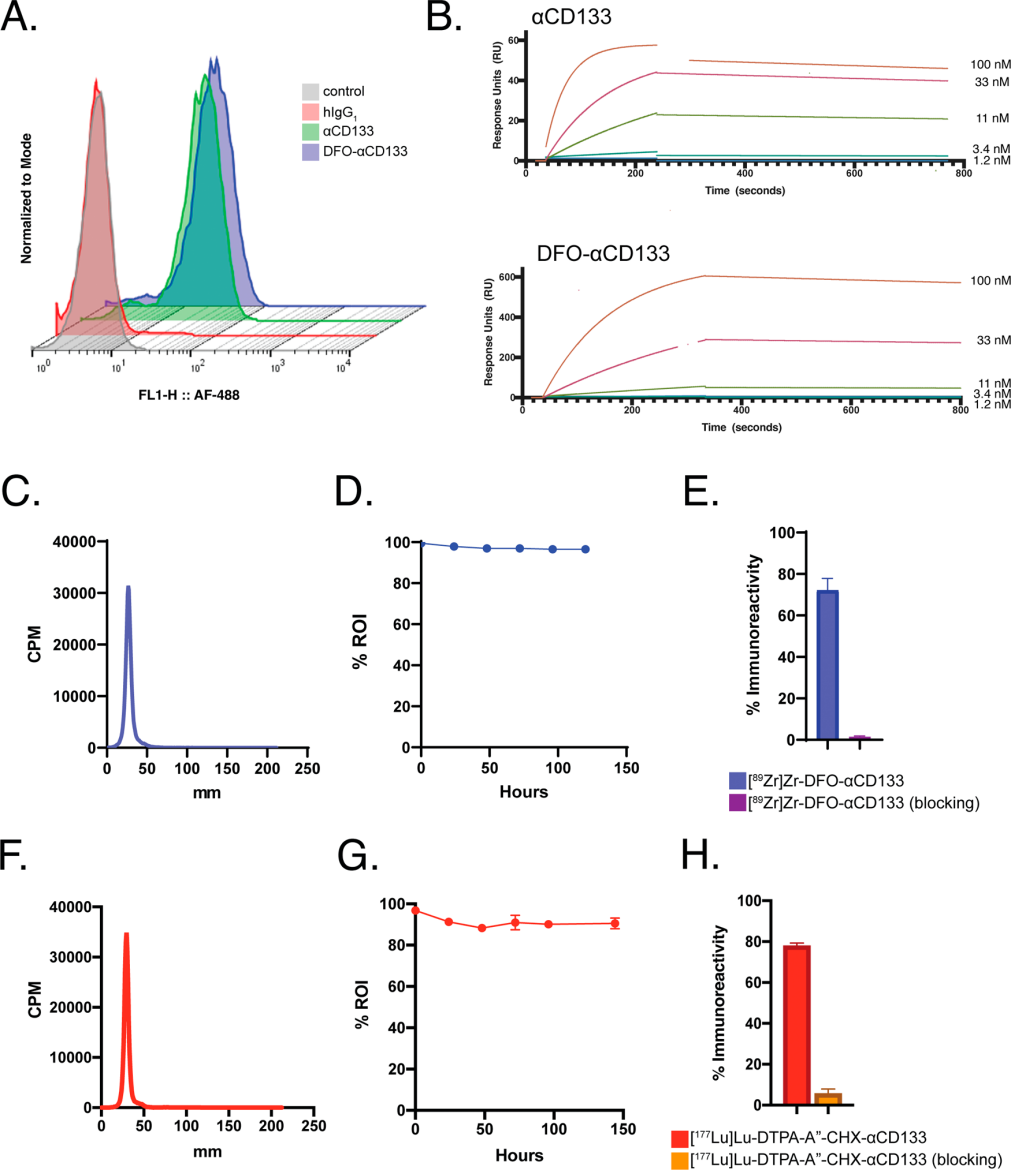
*In vitro* characterization of the radioimmunoconjugates. (A) Flow cytometry of αCD133, DFO-αCD133, and an isotype control mAb with SCLC H82 cells (*n* = 3). Alexa-Fluor 488 was used as the secondary antibody, and a cohort of nonstained cells were used as the control. (B) SPR chromatograms of αCD133 and DFO-αCD133 obtained using immobilized immunoglobulins and varying concentrations of recombinant CD133 as the analyte (1–100 nM). These data correspond with those shown in Table S1. (C) Representative radio-iTLC of [^89^Zr]Zr-DFO-αCD133. (D) Stability assay of [^89^Zr]Zr-DFO-αCD133 upon incubation in human serum for 6 days at 37 °C. (E) Immunoreactivity of [^89^Zr]Zr-DFO-αCD133 performed using CD133-expressing H82 SCLC cells. (F) Representative radio-iTLC of [^177^Lu]Lu-DTPA-A″-CHX-αCD133. (G) Stability assay of [^177^Lu]Lu-DTPA-A″-CHX-αCD133 upon incubation in human serum for 6 days at 37 °C. (H) Immunoreactivity of [^177^Lu]Lu-DTPA-A″-CHX-αCD133 performed using beads coated with recombinant CD133.

DFO-αCD133 was synthesized via the stochastic modification of the lysines of the immunoglobulin with *p*-SCN-Bn-DFO and was obtained in >90% yield following gel filtration and ultracentrifugation. Flow cytometry with CD133-expressing NCI–H82 human SCLC cells revealed that the immunoconjugate’s *in vitro* behavior remained unperturbed compared to that of its parent mAb (Figure 1A). Surface plasmon resonance (SPR) was employed to evaluate the binding of DFO-αCD133 and αCD133 to recombinant CD133 more quantitatively. As expected, the two immunoconjugates exhibited strikingly similar *K*_D_ values (6.92 × 10^–10^ M for αCD133 and 1.58 × 10^–9^ M for DFO-αCD133) and kinetic parameters (Figure 1B and Table S1). After the characterization of the immunoconjugate, DFO-αCD133 was radiolabeled with [^89^Zr]Zr^4+^ using standard protocols to produce [^89^Zr]Zr-DFO-αCD133 in >95% radiochemical yield and a specific activity of 185–740 MBq/mg (5–20 mCi/mg) (Figure 1C).^18^ Stability assays subsequently revealed that the radioimmunoconjugate remained >95% intact after 6 days at 37 °C in human serum (Figure 1D). Finally, a cell-based immunoreactivity assay with NCI–H82 SCLC cells was used to confirm that [^89^Zr]Zr-DFO-αCD133 boasted an immunoreactive fraction of >0.70 (Figure 1E).

Because NSG mice were used instead of nude mice for two of the murine models used in the PET imaging studies, the metastatic and PDXs, one additional structural modification was needed for the immunoconjugate. We have previously reported that NSG mice (which lack endogenous mIgG) exhibit very high uptake of radioimmunoconjugates in the liver and spleen, presumably because circulating and tissue-resident macrophages with unoccupied mFcγRI receptors bind the radiolabeled antibodies and sequester them in these organs.^22,23^ We have found that this phenomenon can be avoided via the deglycosylation of immunoconjugates as the removal of the heavy chain glycans abrogates FcγRI binding, thereby helping radioimmunoconjugates avoid capture by mFcγRI-bearing myeloid cells and reducing their consequent accretion in the spleen and liver.^24,25^ While we do not yet know if this phenomenon extends to human patients, reducing the uptake of the radioimmunoconjugate in the spleen and liver in this way could significantly reduce radiation dose rates to these tissues, thereby preventing side effects such as radiation-induced liver disease.^26^ For this study, DFO-αCD133 was deglycosylated with PNGase F to create ^degly^DFO-αCD133, and the deglycosylation was confirmed via SDS-PAGE (Figure S1). ^degly^DFO-αCD133 was then radiolabeled in a manner identical to its glycosylated parent to produce [^89^Zr]Zr-^degly^DFO-αCD133 in >90% radiochemical yield and a specific activity of 185–740 MBq/mg (5–20 mCi/mg).

### ^89^Zr-ImmunoPET in an Orthotopic Xenograft Model

**Figure 2:**
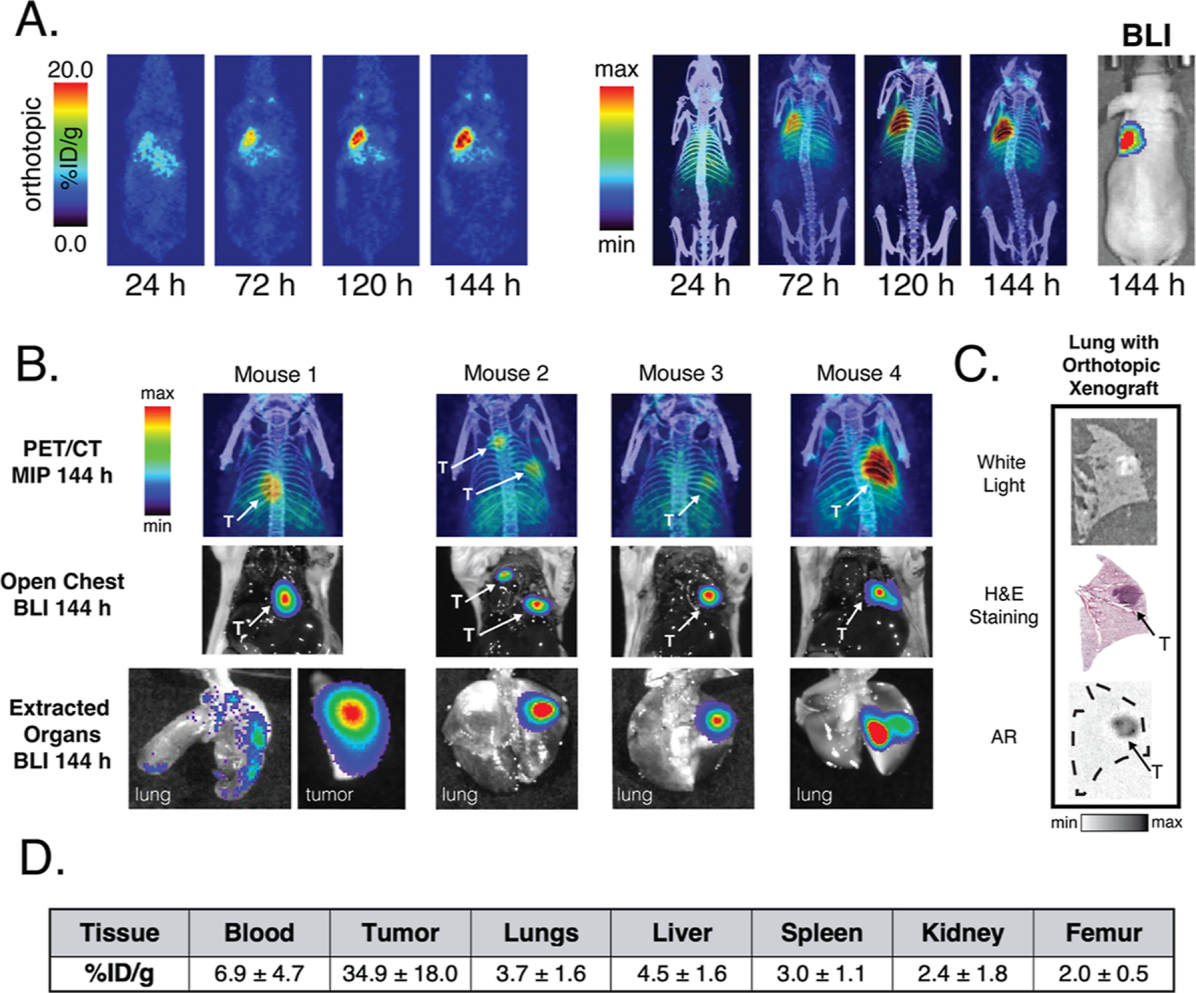
*In vivo* evaluation of [^89^Zr]Zr-DFO-αCD133 in mice bearing orthotopic SCLC xenografts. (A) Coronal PET slices (left) and PET/CT maximum intensity projections (MIPs) (right) of [^89^Zr]Zr-DFO-αCD133 [3.7–3.9 MBq (100–105 μCi), 5–5.5 μg in 100 μL of PBS] in mice bearing orthotopic H82-*luc* xenografts in the left lung. A BLI image collected after the terminal imaging time point (i.e., 144 h) is shown on the far right for comparison. (B) Comparison of PET MIPs to *ex vivo* BLI images in each of the mice. Immediately prior to euthanasia, the mice were injected with D-luciferin, as described in Methods. Following euthanasia, BLI images of the open chest were acquired, followed by additional images of selected extracted organs. Since the open chest BLI images were acquired in an anterior orientation, the PET/CT images in the top row were reflected to better demonstrate correlations. Mouse 1 did not have any tumors that grew in the lung but had a tumor that grew elsewhere in the thoracic cavity. Mouse 2 had both a small tumor growing in the lung and a small tumor growing outside the lung in the upper thoracic cavity. (C) White-light image, hematoxylin and eosin (H&E) staining, and AR of the left (tumor-bearing) lung of mice that had been injected with [^89^Zr]Zr-DFO-αCD133. Tumor lesions are denoted by arrows. (D) Quantitative biodistribution data collected after the final imaging time point at 144 p.i.

The initial goal of this investigation was to build upon our preliminary ^89^Zr-immunoPET imaging experiments in mice bearing subcutaneous xenografts with experiments in more clinically representative murine models of SCLC. To this end, we first focused on an orthotopic model of SCLC predicated on the implantation of luciferase-expressing H82-*luc* cells into the left lungs of mice. The growth of the xenografts was monitored via BLI, and about 25% of the mice developed tumors, with half growing outside of the lungs but still within the thoracic cavity. Once the growth of the tumors was confirmed via BLI, the mice (*n* = 4) were administered [^89^Zr]Zr-DFO-αCD133 [3.7–3.9 MBq (100–105 μCi), 5–5.5 μg, in 100 μL of PBS] via the lateral tail vein, and serial PET images were acquired daily over the course of 6 days (Figures 2 and S2). These images revealed substantial uptake of [^89^Zr]Zr-DFO-αCD133 in the left lungs of the mice over the course of the study, including peak values over 20 %ID/g at 144 h post-injection as well as relatively low activity concentrations in healthy tissues. Critically, orthotopic xenografts as small as <2 mm in diameter could be effectively visualized. After the final imaging time point, the mice were euthanized, and several *ex vivo* experiments were performed to further interrogate the performance of the probe. Post-mortem open-chest BLI revealed a clear correlation between the location of the luminescence signal in the H82-*luc*-bearing left lungs of the mice and the PET data (Figure 2B). Subsequently, histology and autoradiography (AR) of left lung tissue sections reinforced the colocalization of the tumor cells and radioactivity (Figure 2C). Finally, a quantitative biodistribution assay reinforced the imaging data, revealing a terminal tumoral activity concentration of 35 ± 18 %ID/g as well as far lower accretion levels in the liver (4.5 ± 1.6), spleen (3.0 ± 1.1), kidneys (2.4 ± 1.8), bones (2.0 ± 0.5), and blood (6.9 ± 4.7) (Table S2).

### ^89^Zr-ImmunoPET in a Metastatic Xenograft Model

**Figure 3:**
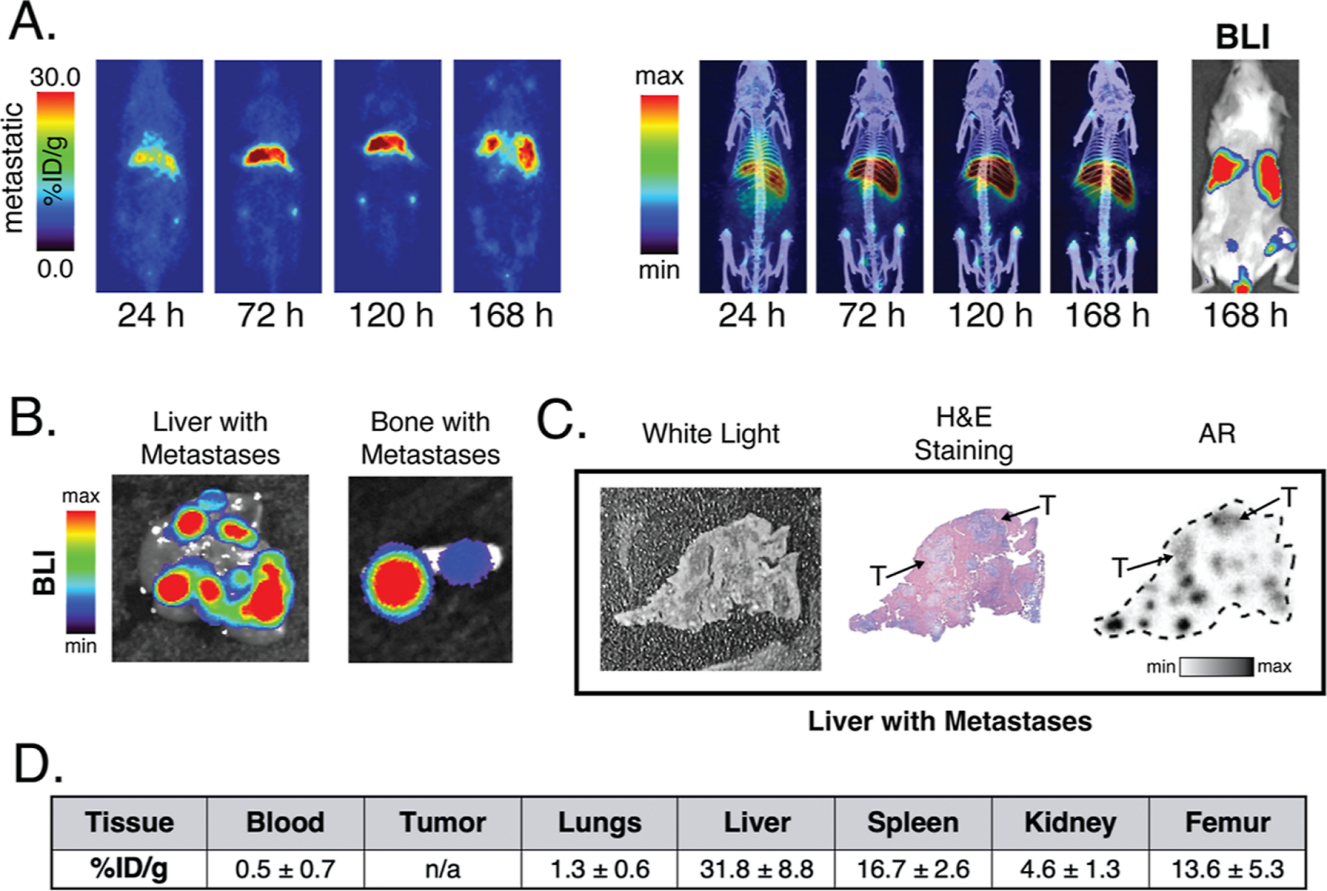
*In vivo* evaluation of [^89^Zr]Zr-^degly^DFO-αCD133 in a metastatic model of SCLC. (A) Coronal PET slices (left) and PET/CT MIPs (right) of [^89^Zr]Zr-^degly^DFO-αCD133 [3.7–3.9 MBq (100–105 μCi), 5–5.5 μg in 100 μL of PBS] in mice bearing metastatic H82-*luc* lesions in the liver and bones. A BLI image acquired after the terminal imaging time point (i.e., 168 h) is shown on the far right. (B) *Ex vivo* BLI images of the liver (left) and bone (right) confirm the presence of tumor tissue. (C) White-light image, H&E staining, and AR of the liver of a mouse that had been injected with [^89^Zr]Zr-^degly^DFO-αCD133. Tumor lesions are denoted by arrows. (D) Quantitative biodistribution data collected after the final imaging time point at 168 p.i.

With these data in hand, we next turned to a metastatic model of SCLC based on the injection of H82-*luc* cells into the tail veins of NSG mice. BLI was used to visualize the growth of SCLC cells in the liver and bones until the lesions reached a suitable size for PET imaging, at which point the mice (*n* = 4) were administered [^89^Zr]Zr-^degly^DFO-αCD133 [3.7–3.9 MBq (100–105 μCi), 5–5.5 μg, in 100 μL of PBS]. Serial PET images were collected over the course of 1 week, revealing uptake in the liver and bones that reached ∼30 and ∼13 %ID/g, respectively, by the end of the experiment as well as low activity concentrations in healthy organs (Figures 3A, S3, and S4). Critically, the PET signal in both the liver and bones correlated with the BLI signal in these tissues, strongly suggesting that the accretion of [^89^Zr]Zr-^degly^DFO-αCD133 was mediated by tumor cells and not normal physiological processes such as the clearance of the radioimmunoconjugate (i.e., in the liver) or the deposition of free [^89^Zr]Zr^4+^ (i.e., in the bones). The *ex vivo* analysis of tissue samples from the mice reinforced the sensitivity of the radiotracer as AR and histology of lung and liver sections clearly demonstrated the microscopic colocalization of the radioactivity and tumor cells within these two tissues (Figure 3B,C). Finally, a terminal biodistribution confirmed the observations from PET. At 168 h post-injection, the tissues containing metastatic lesions showed high activity concentrations: 31.8 ± 8.7% ID/g in the liver (note: the tumor lesions were too small to excise from the liver itself) and 13.6 ± 5.6 %ID/g in the femur. Most healthy tissues exhibited very low levels of uptake, e.g., 1.3 ± 0.6 %ID/g in the lungs and 0.5 ± 0.7 %ID/g in the blood, but elevated levels of radioactivity could be seen in the spleen (16.7 ± 2.6 %ID/g), perhaps an artifact of the mouse model itself (Figure 3D and Table S2).

### ^89^Zr-ImmunoPET in a PDX Model

**Figure 4:**
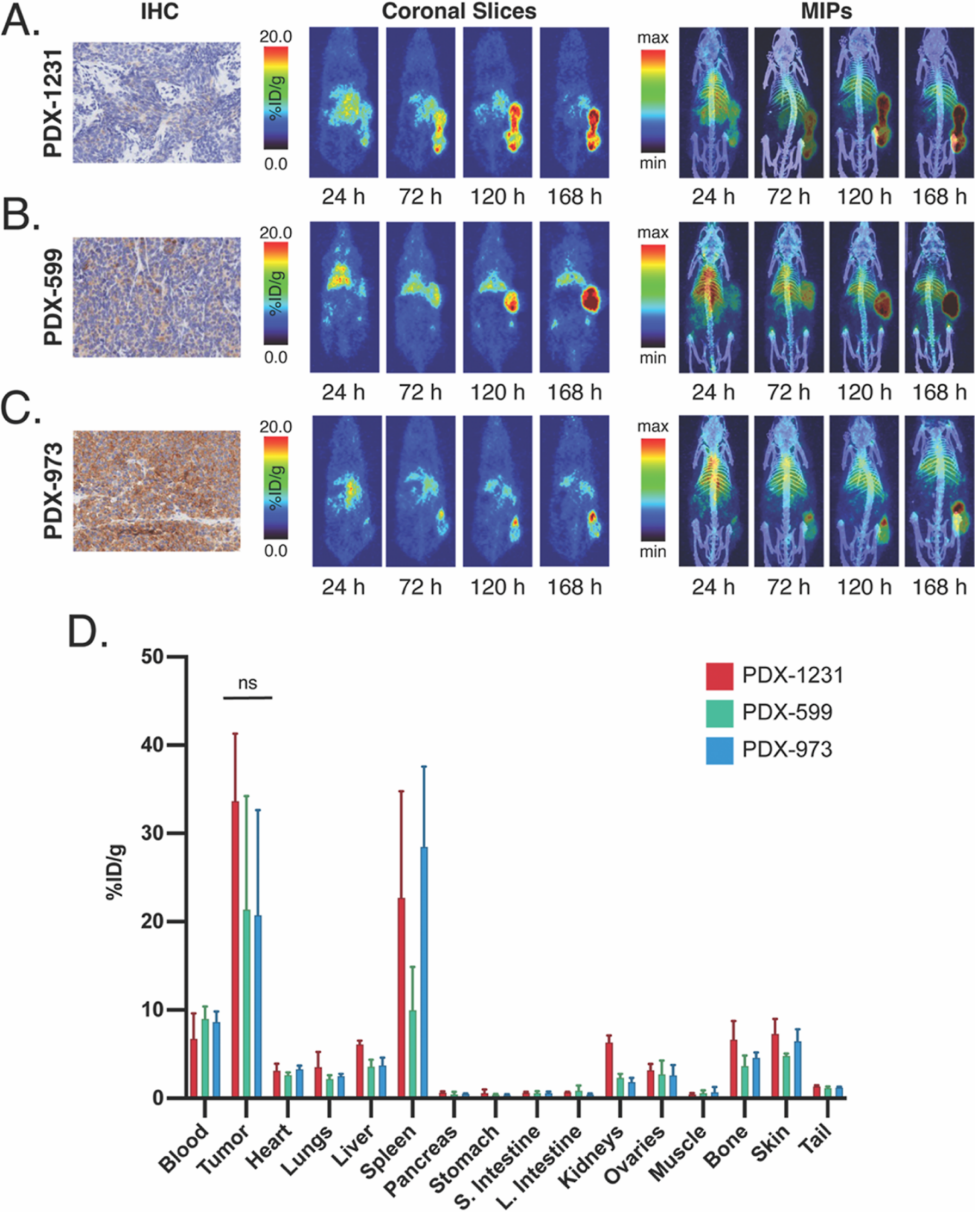
*In vivo* evaluation of [^89^Zr]Zr-^degly^DFO-αCD133 in mice bearing subcutaneous SCLC PDXs. (A–C) PET and PET/CT images acquired after the administration of [^89^Zr]Zr-^degly^DFO-αCD133 [3.7–3.9 MBq (100–105 μCi), 10–10.5 μg in 100 μL of PBS] to mice bearing PDX-1231 (top), PDX-599 (middle), and PDX-973 (bottom) xenografts as well as the immunohistochemical staining of slices of each PDX for CD133. (D) Quantitative biodistribution data collected after the final imaging time point at 168 p.i. Statistical differences were determined using an unpaired, two-tailed student’s test (with Welch’s correction) using GraphPad Prism 7.0.

The final set of experiments in our evaluation of ^89^Zr-labeled αCD133 utilized a trio of patient-derived SCLC xenografts—PDX-1231, PDX-599, and PDX-973—obtained from the Anti-Tumor Assessment Core of MSKCC. After immunohistochemistry (IHC) was used to confirm their expression of CD133, the three PDXs were implanted subcutaneously into the flanks of NSG mice (*n* = 4 per PDX). Once the tumors reached ∼100 mm^3^, the mice were intravenously administered [^89^Zr]Zr-^degly^DFO-αCD133 [3.7–3.9 MBq (100–105 μCi), 5–5.5 μg, in 100 μL of PBS], and PET images were acquired daily over the course of 1 week (Figures 4A–C and S5–S7). As early as day 2, a high tumor-to-background contrast was observed in all of the mice. High concentrations of the radioimmunoconjugate accumulated in the PDX-1231, PDX-599, and PDX-973 xenografts by the end of the experiment (>20 %ID/g by 168 h post-injection). A terminal biodistribution study reinforced the imaging results, revealing activity concentrations of 33.6 ± 7.6, 21.4 ± 12.8, and 20.7 ± 11.9 %ID/g in the PDX-1231, PDX-599, and PDX-973 xenografts, respectively, at 168 h post-injection (Figure 4D and Table S3). Generally speaking, far lower levels of uptake were seen in healthy tissues, with most showing activity concentrations under 5 %ID/g. With uptake values above 10 %ID/g in each of the cohorts, the spleen stood as the lone exception to this trend, although this is most likely physiological accretion related to the strain of the mouse (i.e., NSG) and not the presence of the xenografts. It was slightly surprising that PDX-973—which seemed to boast the highest expression of CD133 via IHC—did not exhibit higher uptake of the radioimmunoconjugate than that of the other two tumors. While the explanation for this phenomenon is likely multiparametric, we hypothesize that it could stem from differences in the perfusion and stromal density of the three xenografts.

### Radioimmunotherapy

The efficacy of [^89^Zr]Zr-DFO-αCD133 as a PET imaging agent led us to investigate CD133 as a target for the radioimmunotherapy of SCLC, a particularly promising application given the documented radiosensitivity of the disease.^27^ To this end, we first synthesized a variant of the antibody bearing a chelator, CHX-A″-DTPA, that stably coordinates the β-emitting radiometal ^177^Lu. More specifically, DTPA-A″-CHX-αCD133 was synthesized via the stochastic bioconjugation of *p*-SCN-Bn-CHX-A″-DTPA to the lysines of αCD133, ultimately providing the final immunoconjugate in >90% yield following gel filtration and ultracentrifugation. DTPA-A″-CHX-αCD133 was subsequently incubated with [^177^Lu]Lu^3+^ according to literature protocols, affording [^177^Lu]Lu-DTPA-A″-CHX-αCD133 in >95% radiochemical yield and specific activities of 74–370 MBq/mg (2–10 mCi/mg) (Figure 1F).^28^ Stability assays demonstrated that [^177^Lu]Lu-DTPA-A″-CHX-αCD133 remained >90% intact after 6 days at 37 °C in human serum (Figure 1G). Finally, a bead-based immunoreactivity assay confirmed that [^177^Lu]Lu-DTPA-A″-CHX-αCD133 had an immunoreactive fraction of >0.70 (Figure 1H).

The longitudinal therapy study was performed in mice bearing subcutaneous NCI–H82 xenografts, a murine model that is a suboptimal recapitulation of human disease but nonetheless lends itself to evaluation of therapeutic efficacy due to the ease and quantitative accuracy of the measurement of tumor burden (i.e., volume). Once the xenografts reached a volume of 100 mm^3^, two control cohorts (*n* = 10 mice each) received either saline or 50 μg of unlabeled cold DTPA-A″-CHX-CD133. Dosimetry calculations performed using data from [^89^Zr]Zr-DFO-αCD133 PET images were used to select two doses of [^177^Lu]Lu-DTPA-A″-CHX-αCD133 for the therapy cohorts (*n* = 10 mice each): 125 μCi (4.6 MBq; 50 μg) and 250 μCi (9.3 MBq; 50 μg) (Table S4). Tumor volumes were measured twice per week, and blood was collected once per week for hematoxicology analysis. The study had three predetermined end points: (i) if the mouse lost or gained >10% of its body weight between measurements, (ii) if the volume of the tumor exceeded >2000 mm^3^, and/or (iii) if the mouse became lethargic or skeletal in appearance.

**Figure 5:**
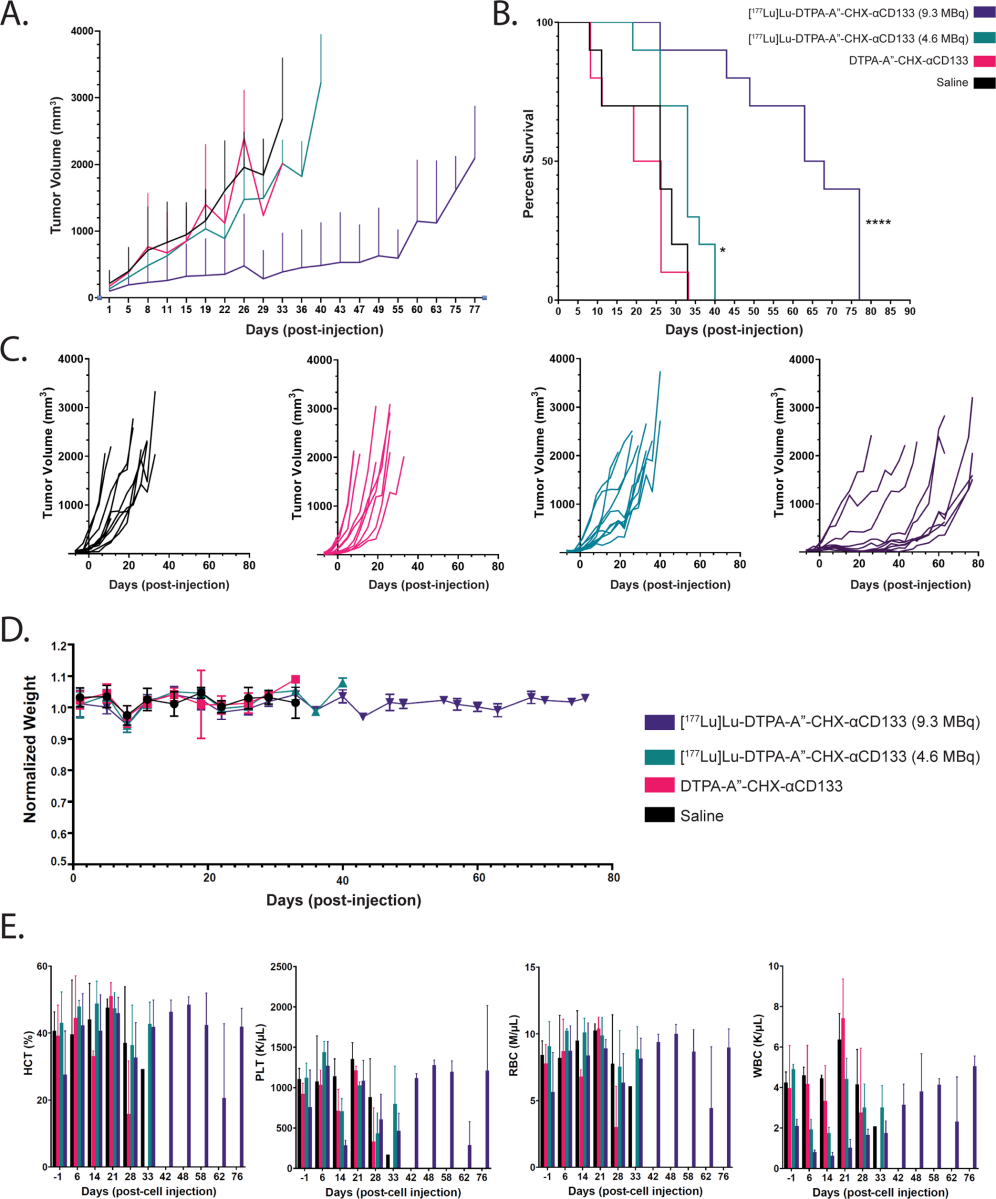
Longitudinal radioimmunotherapy study with [^177^Lu]Lu-DTPA-A″-CHX-αCD133 in mice bearing subcutaneous H82 xenografts. (A) Average tumor volumes as a function of time. (B) Kaplan–Meier plot depicting the survival of mice in each cohort. Median survivals = 22.5 days (saline), 26 days (DTPA-A″-CHX-αCD133), 33 days ([^177^Lu]Lu-DTPA-A″-CHX-αCD133 125 μCi (4.6 MBq)), and 65.5 days ([^177^Lu]Lu-DTPA-A″-CHX-αCD133 250 μCi (9.3 MBq)). (C) Tumor volumes of the individual mice in each cohort as a function of time. The significance analyses were performed with GraphPad Prism 7.0 software by using the log-rank Mantel–Cox test. (D) Normalized body weight of the mice of each cohort. (E) Hematological counts (*n* = 1–3) for the mice in the longitudinal therapy study. HCT: hematocrit. RBC: red blood cell count. PLT: platelet count. WBC: white blood cell count.

The longitudinal therapy study clearly revealed a dose-dependent therapeutic effect for radioimmunotherapy with [^177^Lu]Lu-DTPA-A″-CHX-αCD133 (Figure 5A–C). The two control groups, saline and unlabeled DTPA-A″-CHX-αCD133, showed unchecked tumor growth alongside median survival times of 22.5 and 26 days, respectively. The low-dose cohort [4.6 MBq (125 μCi)] was likewise characterized by rapid tumor growth and had a median overall survival time (33 days) that was statistically significantly increased compared to that of the group treated with saline only (*p ≤ 0.05 via log-rank Mantel–Cox test) but not when compared to that of the cohort treated with the unlabeled immunoconjugate. Critically, however, the high-dose cohort [9.3 MBq (250 μCi)] boasted significantly slowed tumor growth and a median overall survival time of 65.5 days (****p ≤ 0.0001 via log-rank Mantel–Cox test), underscoring the potential of CD133 as a target for radiopharmaceutical therapy.

In order to probe the potential toxicity of the radioimmunoconjugates, both the weights and hematological parameters—i.e., hematocrit (HCT), platelet (PLT), red blood cell (RBC), and white blood cell (WBC) counts—of the mice were monitored throughout the therapy study (Figure 5D,E). Generally speaking, the weights of the mice remained within the prescribed ±10% range. Indeed, only two mice—one from the saline group and one from the unlabeled DTPA-CHX-A″-αCD133 cohort—were euthanized due to having a necrotic tumor (the former, at day 11) or losing over 10% of their body weight (the latter, at day 19). All other mice were euthanized because their tumors exceeded our prescribed maximum volume. The hematological analyses were slightly more telling. Immediately following the injection of [^177^Lu]Lu-DTPA-CHX-A″-αCD133, significant decreases in WBC and PLT levels were observed in both the low- and high-dose cohorts. However, these values gradually recovered to normal levels over the course of 1 month (∼33 days). Finally, none of the mice demonstrated significant behavioral or physical traits that indicate toxicity from either the immunoconjugate or radioimmunoconjugate (Table S5).

Taken together, these data underscore that RIT with [^177^Lu]Lu-DTPA-CHX-A″-αCD133 could be a safe and effective approach to the treatment of SCLC. However, it is important to consider two caveats. First, the regrowth of the tumors even in the high-dose cohort suggests that the clinical efficacy of [^177^Lu]Lu-DTPA-A″-CHX-αCD133 may depend upon using higher doses, adopting a fractionated dosing schema, or combining the radioimmunoconjugate with chemo- or immunotherapeutics.^29−32^ Second, the expression of CD133 by human stem cells raises the spectre of hematopoietic toxicity in patients, and the hematological data from the murine therapy study support the notion that doses may need to be carefully selected in the clinic. If hematotoxicity proves to be a concern, strategies such as fractionated dosing and *in vivo* pretargeting may be adopted to reduce radiation dose rates to healthy tissues.^33,34^ Of course, the translational relevance of either issue cannot be truly known until biodistribution and dosimetry data from patients are obtained in a pilot, first-in-human study.

## Conclusions

The preclinical *in vivo* data described herein clearly indicate the promise of CD133 as a radiotheranostic target for SCLC: [^89^Zr]Zr-DFO-αCD133 delineated tumor tissue with high contrast in several advanced murine models of human disease, while [^177^Lu]Lu-DTPA-CHX-A″-αCD133 exerted a clear dose-dependent therapeutic effect. However, it is nonetheless important to address the limitations of this work. First, the intrinsic heterogeneity of SCLC means that CD133 is unlikely to be a viable target in all cases of the disease; indeed, our previous work illustrated that CD133 was expressed—both at the transcript and protein levels—in ∼60% of SCLC cases.^17^ As a result, it is likely that serum autoantibody assays such as those described in our initial investigation will be highly valuable prior to initiating CD133-targeted immunoPET and radioimmunotherapy. Second, the relatively sluggish pharmacokinetic profiles of full-length radioimmunoconjugates have historically presented challenges in the context of radioimmunotherapy due to their tendency to produce high radiation doses to healthy tissues, such as the kidneys and red marrow. As a result, the use of alternative platforms or approaches that offer more rapid pharmacokinetic profiles and/or higher therapeutic indices—such as antibody fragments or *in vivo* pretargeting—may increase the likelihood of safety and efficacy in the clinic. In the near future, it is our hope to both expand our investigations into radiotheranostics for SCLC by working to create CD133-targeted probes with more rapid *in vivo* profiles and pursue other promising targets for the nuclear imaging and radiopharmaceutical therapy of this devastating disease.

## Supporting Information Available

Additional experimental methods, SDS-PAGE results, additional PET and BLI images, kinetic and thermodynamic binding parameters, biodistribution data, dosimetry values, and survival data for the longitudinal therapy study (PDF)

## Abbreviations

SCLC: small-cell lung cancer
PET: positron emission tomography
RIT: radioimmunotherapy
mAb: monoclonal antibody
*p*-SCN-DFO: 1-(4-isothiocyanatophenyl)-3-[6,17-dihydroxy-7,10,18,21-tetraoxo-27-(*N*-acetylhydroxylamino)-6,11,17,22-tetraazaheptaeicosine] thiourea
*p*-SCN–CHX-A″-DTPA: [(R)-2-amino-3-(4-isothiocyanatophenyl)propyl]-trans-(*S*,*S*)-cyclohexane-1,2-diamine-pentaacetic acid
CT: computed tomography
PBS: phosphate-buffered saline
MWCO: molecular weight cutoff
EDTA: ethylenediaminetetraacetic acid
iTLC: instant thin layer chromatography
hIgG: antihuman immunoglobulin G antibody
%ID/g: percentage injected dose per gram of tissue
MSKCC: Memorial Sloan Kettering Cancer Center
PDX: patient-derived xenograft
SE-HPLC: size exclusion high-performance liquid chromatography
FACS: fluorescence-activated cell sorting
IACUC: institutional animal care and use committee
BLI: bioluminescence imaging
H&E: hematoxylin and eosin
AR: autoradiography
IHC: immunohistochemistry
