# Supplementary material for: Evaluating CD133 as a Radiotheranostic Target in Small-Cell Lung Cancer
Source: Mol Pharm. 2024 Feb 8;21(3):1402–13. doi: 10.1021/acs.molpharmaceut.3c01063 (PMC10915790; doi:10.1021/acs.molpharmaceut.3c01063)
Supplement: Supplementary file 1 — mp3c01063_si_001.pdf [file mp3c01063_si_001.pdf]

Supporting Information

Evaluating CD133 as a Radiotheranostic Target in Small Cell Lung Cancer

Samantha M. Sarrett[1,2,3], Cindy Rodriguez[1,3,4], Samantha Delaney[1,2,3], Meena M. Hosny[1], Joni Sebastiano[1,2,3], Ana Santos Coquillat[5], Outi M. Keinänen[1,3,6], Lukas M. Carter[3], Kristy J. Lastwika[7,8], Paul D. Lampe[9,10], Brian M. Zeglis[1,2,3,4]*

[1]Department of Chemistry, Hunter College of the City University of New York, New York, NY, 10065, USA

[2] Ph.D. Program in Biochemistry, The Graduate Center of the City University of New York, New York, NY, 10016, USA

[3]Department of Radiology, Memorial Sloan Kettering Cancer Center, New York, NY, 10065, USA

[4]Ph.D. Program in Chemistry, The Graduate Center of the City University of New York, New York, NY, 10016, USA

[5]Department of Chemistry, CICECO – Aveiro Institute of Materials, University of Aveiro, Campus Universitario de Santiago, Aveiro, 3810-193, Portugal

[6]Department of Chemistry, University of Helsinki, Helsinki, 00100, Finland

[7]Translational Research Program, Public Health Sciences Division, Fred Hutchinson Cancer Research Center, Seattle, Washington, 98109, USA

[8]Clinical Research Division, Fred Hutchinson Cancer Research Center, Seattle, Washington, 98109, USA

[9]Human Biology Division, Fred Hutchinson Cancer Research Center, Seattle, Washington, 98109, USA

[10]Translational Research Program, Public Health Sciences Division, Fred Hutchinson Cancer Research Center, Seattle, Washington, 98109, USA

***Corresponding Author:** Brian M. Zeglis; 413 East 69th Street, New York, NY, 10021; Phone: 212-896-0433; E-mail: bz102@hunter.cuny.edu

Running Title: CD133-Targeted Radiotheranostics in SCLC

Keywords: PET Imaging, Radioimmunotherapy, Orthotopic Xenograft, Metastatic Xenograft, Patient-Derived Xenograft, CD133, Small Cell Lung Cancer

SUPPLEMENTAL METHODS

Serum Stability Studies

The stabilities of the radioimmunoconjugates were interrogated by incubating [^{89}Zr]Zr-DFO-αCD133 or [^{177}Lu]Lu-DTPA-A"-CHX-αCD133 in human serum on a ThermoMixer at 500 rpm and 37 °C for 6 days. Every 24 h, the radiochemical purity of the radioimmunoconjugates were determined in triplicate via radio-iTLC measurements with an eluent of 50 mM EDTA, pH 5.0.

Immunoreactivity Assays

Cell Saturation Assay with DFO-αCD133

The immunoreactivity of [^{89}Zr]Zr-DFO-αCD133 was determined using a cell saturation assay. Briefly, 15 × 10^6 H82 (CD133+) cells were washed 3× with ice-cold media, centrifuged (600 g, 2 min), and the supernatant was discarded. Ice-cold media (200 µL) with 1 µL of [^{89}Zr]Zr-DFO- αCD133 (1 µg/mL in Chelex-PBS + 1% BSA, pH 7.4) was added to the cell pellet, mixed thoroughly, and allowed to incubate for 1 h. After the incubation period, the cells were centrifuged, and the supernatant was reserved. The cells were washed 2× with ice-cold media and the supernatants were each reserved in a separate microcentrifuge tube. The samples were then measured on an ^{89}Zr-calibrated gamma counter, with the activities (counts/minute) background- and decay-corrected to the start of the run. The immunoreactivity was expressed as a percentage by comparing the activity remaining in the cells to the total activity (cells + supernatant + washes). For the blocking experiments, the assay was run identically, but 5 µg of unlabeled αCD133 were co-incubated with the cells along with [^{89}Zr]Zr-DFO-αCD133.

Bead-Based Assay with DTPA-A"-CHX-αCD133

For the DTPA-conjugated mAb, a bead-based immunoreactivity assay was used as described by Sharma, *et al.*.[1] Briefly, 20 µL of HisPur™ Ni-NTA magnetic beads were washed twice with PBS + 0.05% Tween-20 (PBS-T). After each wash, the tubes were placed on a magnetic rack and the supernatant was discarded. Next,

200 μL PBS-T + 10 μL CD133 antigen (0.1 mg/mL in Chelex-PBS + 1% BSA, pH 7.4) were added to the beads and the solutions were mixed thoroughly. The tubes were incubated for 15 min at room temperature on a rotating platform. Following incubation, the beads were washed twice with PBS-T. One cohort of beads did not receive any antigen to serve as a negative control. Next, 1 ng of [^{177}Lu]Lu-DTPA-A"-CHX-αCD133 (1 μg/mL in Chelex-PBS + 1% BSA, pH 7.4) was added. The samples were allowed to react for 30 min on a rotating platform at room temperature. Following incubation, the supernatants were collected. The beads were then washed twice with PBS-T, and the supernatants of each wash were collected. All samples were then measured on a ^{177}Lu-calibrated gamma counter, with the activities (counts/minute) background- and decay-corrected to the start of the run. The immunoreactivity was expressed as a percentage by comparing the activity remaining in the beads to the total activity (beads + supernatant + washes) (n = 3). Blocking studies were performed as described in the cell saturation assay above.

Surface Plasmon Resonance

The affinity of αCD133 for the CD133 antigen was measured using surface plasmon resonance experiments. Briefly, protein A was immobilized onto an activated carboxyl sensor using Nicoya OpenSPR kit as per the manufacturer's instructions. The mAb — αCD133 or DFO-αCD133 diluted in running buffer (HBS + 0.05% P-20 + 0.1% BSA) — was captured onto the protein A sensor (25 μg/mL over 300 s). A multicycle kinetics experiment was performed by flowing 1.23, 3.4, 11, 33, and 100 nM HER2 antigen solutions (prepared in running buffer) over the sensor for 300 s. Glycine HCl (10 mM, pH 1.5) was used as a regeneration solution between each antigen injection to strip the mAb from the protein A prior to the subsequent antigen concentration injection. Blank buffer runs were subtracted from the results and the kinetics were determined using TraceDrawer.

Preparation of Tissue Slides

<u>From the Orthotopic Xenograft Model</u>

Following the final PET imaging timepoint, the orthotopic mice were sacrificed via $CO_{2(g)}$ asphyxiation. The lungs were first perfused through the right ventricle of the heart with 3 mL PBS using a 28-gauge needle. Then, the lungs were inflated with ~2 mL of a 50:50 formalin:OCT mixture via a 23-gauge needle inserted into the trachea. The inflated lungs were immediately submerged in formalin and allowed to incubate for 24 h. Afterwards, the lungs were washed with 20% sucrose overnight, and the left and right lungs were separated. The left lungs and the inferior lobe of the right lungs were then embedded in a cryomold with OCT and immediately cryogenically frozen. The next day, 10 μm slices of the tissue were cut using a cryostat microtome and collected onto slides.

From the Metastatic Xenograft Model

Following the final PET imaging timepoint, the metastatic mice were sacrificed via $CO_{2(g)}$ asphyxiation. A portion of the left liver lobe was removed from the mouse, rinsed with water, and dried thoroughly with a paper towel. The livers were then embedded in a cryomold with OCT and flash-frozen with dry ice. The next day, 10 μm slices of the tissue were cut using a cryostat microtome and collected onto slides.

Bioluminescence Imaging

To monitor the growth of the orthotopically and metastatically implanted H82-*luc* cells, bioluminescence images of the mice were collected using an IVIS Spectrum-CT instrument. To this end, 100 μL of 30 mg/mL firefly D-luciferin (IVISBrite Xenolight) in PBS was administered to the mice via an intraperitoneal injection. Subsequently, the mice were anesthetized with 2% isoflurane/$O_{2(g)}$. At 15 min post-injection, the mice were imaged in the prone and lateral positions. All images were analyzed with Living Image®.

Autoradiography of Tissue Slides

Following the protocols described in *Preparation of Tissue Slides*, the slides were placed into a cassette with a clean phosphor imager plate (FujiFilm Imaging Plate, BAS-MS) and the cassette was stored in the dark for 48 h. Following this, the radioactivity on the phosphor imager plate was scanned using Typhoon FLA 7000

instrumentation.

H&E Staining of Tissue Slides

Histology was performed on the tissue slides using a hematoxylin and eosin staining kit from Abcam (ab245880). The tissue slides were fixed with formalin prior to the staining; the lungs were fixed immediately after *ex vivo* extraction, and the flash-frozen liver slides were fixed in a Coplin jar prior to staining. To begin, the slides were rinsed with PBS for 3 min to remove the OCT and air dried. The slides were dipped in the hematoxylin stain (2 min for the lung tissue slides; 4 min for the liver tissue slides), followed by 3 washes in DI water. Then, the slides were dipped in bluing reagent for 10-15 s, followed by two DI water washes. After the second wash, the slides were quickly dipped in 100% ethanol and air dried. Finally, the slides were stained with the eosin (2 min for the lung tissue slides; 1 min for the liver tissue slides) and washed 3× with 100% ethanol. After the slides had completely air dried, they were mounted and sealed with a coverslip. Images were acquired by the Memorial Sloan Kettering Cancer Center Imaging and Image Analysis Core.

Dosimetry

Human/murine organ time-integrated activity coefficients (TIACs units of h or MBq×h/MBq) for [^{177}Lu]Lu-DTPA-A"-CHX-αCD133 were estimated from the PET images of [^{89}Zr]Zr-DFO-αCD133 in mice bearing subcutaneous H82 xenografts (see *PET Imaging* methods in the main text).

The %ID/g murine organ uptake values were converted to standardized uptake values (SUVs; normalized by total body mass) for estimation of organ-level absorbed doses a murine computational phantom. The percentage of injected dose in phantom organ I, $\%ID_I$, was obtained from the equation below, which assumes SUV is independent of body mass:

$$\%ID_I = SUV_i \times \frac{m_I}{m_{TB}} \times 100\%$$

where SUV_i is the measured standardized uptake value for mouse organ i, m_I is the mass of corresponding phantom organ I, and m_{TB} is the total phantom mass. The $\%ID_I$ at each timepoint was subsequently multiplied

by a corresponding radioactive decay factor. To obtain TIACs, the resultant activity-time curves were integrated by the trapezoidal method over interval spanning the time-of-injection to the last measured timepoint (144 h); beyond the last measured timepoint, clearance was assumed to occur via radioactive decay only, and the analytical expression for the integral was used. The TIAC for the rest-of-body was obtained by subtracting the individual organ TIACs from that of the total body. The TIAC for the total body was computed as $t_{1/2}/\ln(2)$ (*i.e.* under the assumption that no biological excretion occurred), where $t_{1/2}$ is the physical half-life of the radionuclide.

Using the derived TIACs, normal organ absorbed dose coefficients were computed for the 25 g reference mouse (MOBY phantom) using PARaDIM 1.0/PHITS version 3.20. To enable tumor absorbed dose estimates, the phantom was modified to include a 100 mm^3 tumor on the left flank.

Gel Electrophoresis

αCD133, DFO-αCD133, and ^{degly}DFO-αCD133 samples were prepared for SDS-PAGE according to the manufacturer's instructions (NuPAGE™, ThermoFisher). Briefly, 2 µg of each antibody were reduced using 10× reducing agent, denatured using 4× LDS buffer, and diluted with DI water. The reduced antibody samples were then placed on a ThermoMixer at 85 ºC and 300 rpm for 15 min. Next, 20 µL of each sample was added to the wells of a Novex 4–12% SDS Page Gel, and the gel box was filled with NuPAGE™ MOPS running buffer. A Novex™ Sharp pre-stained protein ladder was also added on each side of the samples. The gel was allowed to run at 70 V for 2.5 hours and washed 3× with DI water. Enough SimplyBlue™ SafeStain was added to cover the gel, and the gel was allowed to stain for 90 minutes on a shaker. Following staining, the gel was washed 3× with DI water. Finally, the gel was imaged using a LI-COR Odyssey CLx instrument and analyzed using Image Studio™ Acquisition Software.

Flow Cytometry

2×10^6 H82 cells were aliquoted per sample and washed 3× (650 rcf, 2.5 min) with ice-cold PBS. 50

μL of either αCD133, DFO-αCD133, or a non-specific hIgG1 isotype control (6 μg/mL) were added to their respective tubes (n = 3). The cells were incubated on ice for 30 min and washed 3× with ice-cold PBS. After the final wash, 50 μL of goat anti-human IgG Alexa Fluor™ 488 antibody (6 μg/mL) were added to the tubes and the samples were incubated for 30 min on ice. The samples were then washed 3× with ice-cold PBS and the cell pellets were resuspended in FACS buffer (PBS + 0.05% FBS + 2 mM EDTA). Finally, all samples were measured using FACS Caliber instrumentation and the data was analyzed using FlowJo™ software. All samples were performed in triplicate. Cells that did not receive any primary or secondary antibody were used as a non-stained control.

SUPPLEMENTAL FIGURES

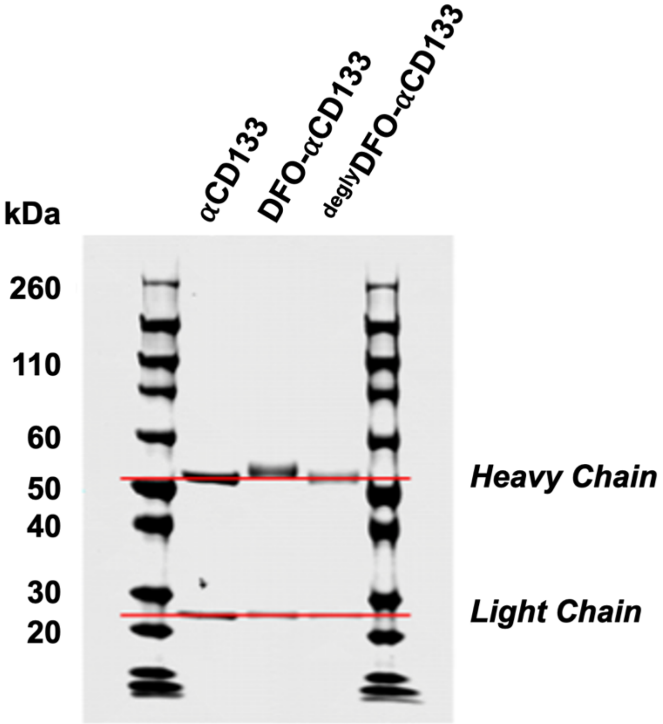

Figure S1. Reducing SDS-PAGE gel of αCD133, DFO-αCD133, and ^{degly}DFO-αCD133.

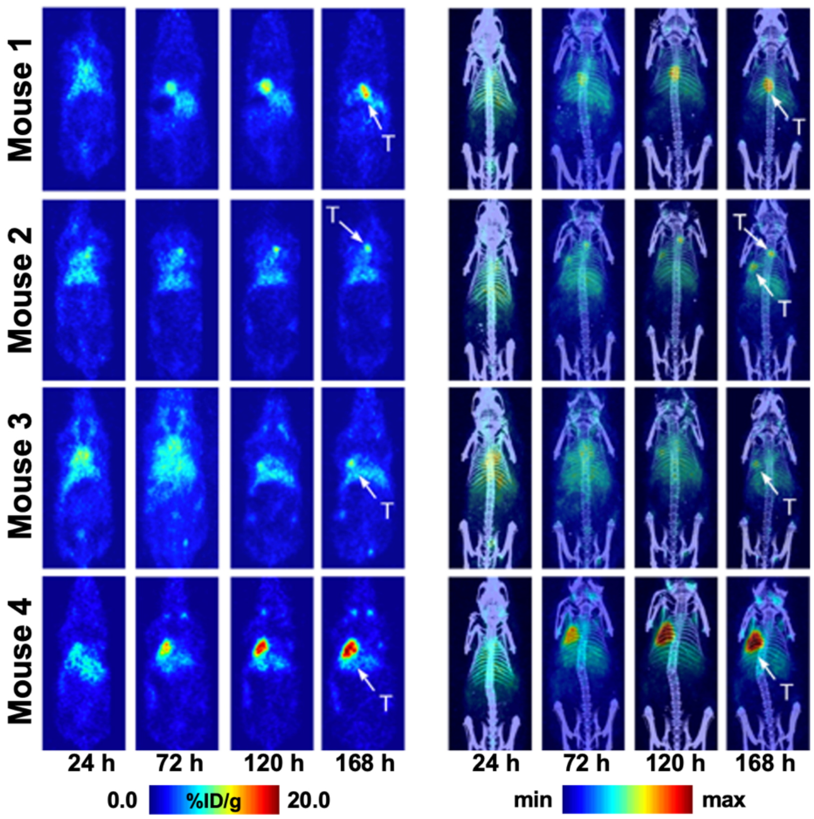

Figure S2. PET images of [^{89}Zr]Zr-DFO-αCD133 [3.7–3.9 MBq (100–105 μCi), 5–5.5 μg in 100 μL of PBS] in mice bearing orthotopic SCLC H82 xenografts (n = 4). Coronal slices are shown on the left and maximum intensity projections (MIPs) are shown on the right. These data correspond with that described in **Table S2**.

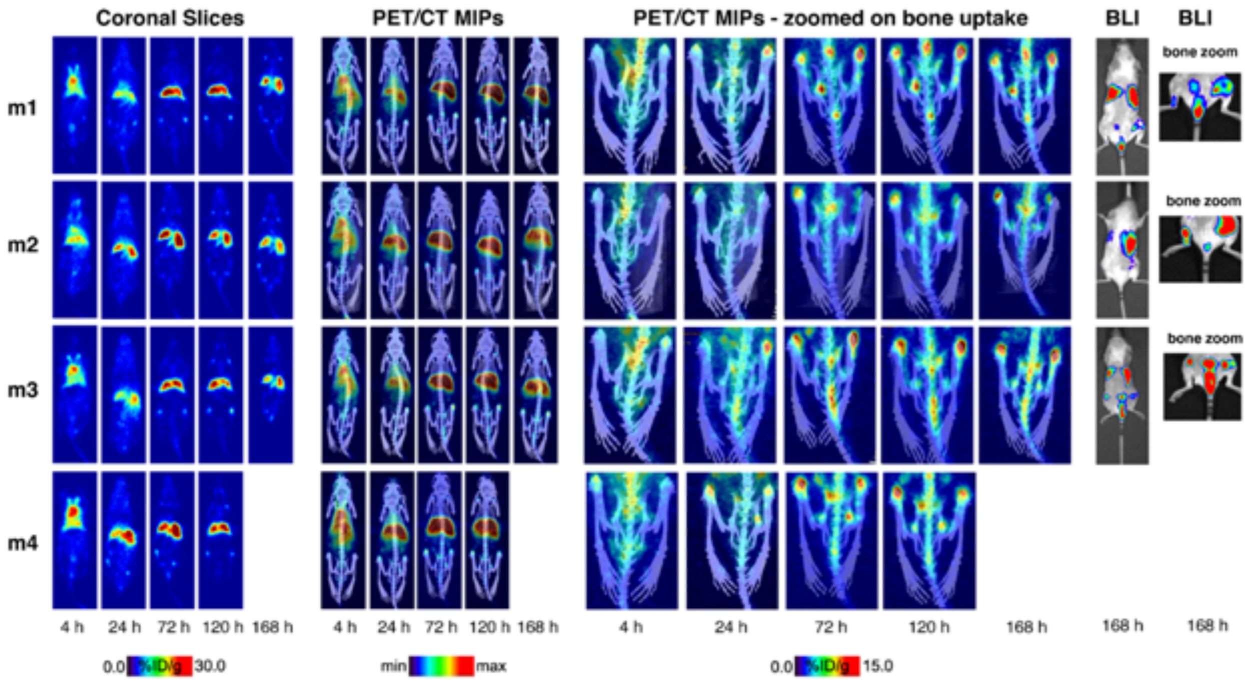

Figure S3. PET and BLI images of [^{89}Zr]Zr-DFO-αCD133 in a metastatic model of SCLC H82 xenografts (n = 4). Mice were injected with 1×10^6 H82-*luc* cells, and SCLC tumor lesions grew in the liver and bones (as observed via BLI imaging). After the confirmation of tumor growth, the mice were then injected with [^{89}Zr]Zr-^{degly}DFO-αCD133 [3.7–3.9 MBq (100–105 μCi), 5–5.5 μg in 100 μL of PBS], and PET images were acquired over the course of 1 week. Coronal slices are shown on the far left, while MIPs (overlayed with CTs) are shown in second from the left. Zoomed-in images delineating the uptake of the radioimmunoconjugate in the bone are shown second to the right, and BLI images acquired immediately after the final PET imaging time point are shown on the far right. Mouse 4 (m4) unexpectedly died during the collection of the 120 h PET image and is not shown thereafter. These data correspond with that described in **Table S2**.

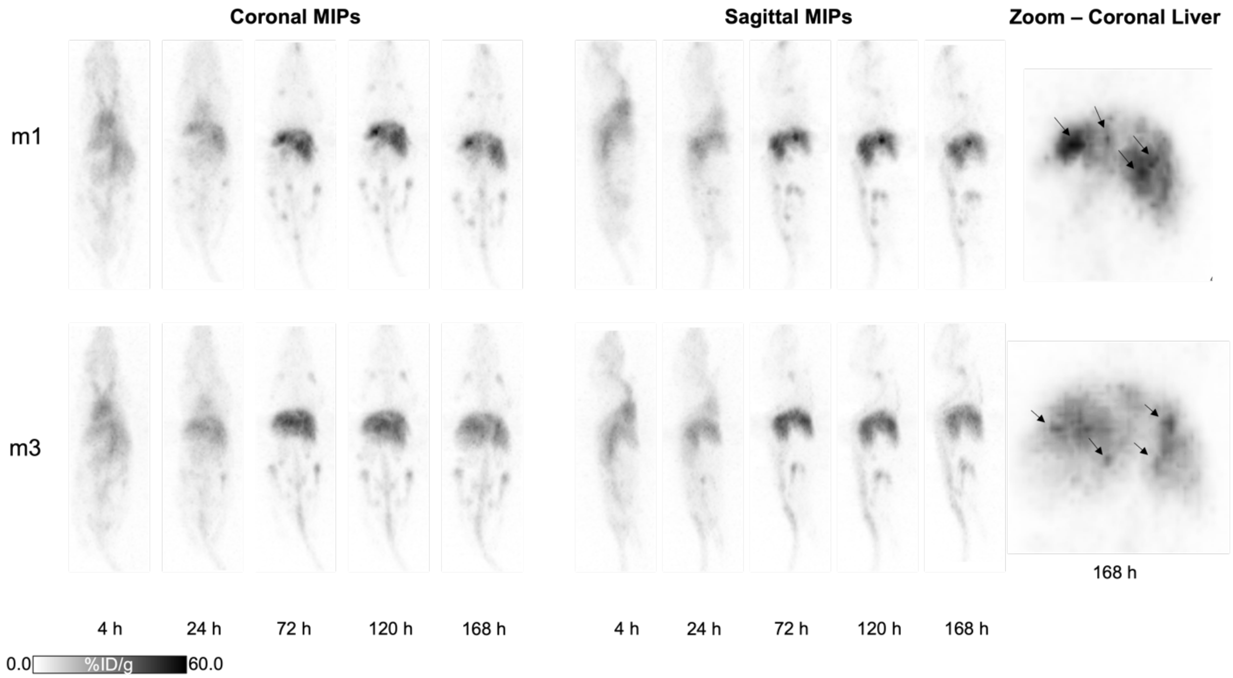

Figure S4. Representative PET MIPs of two mice bearing metastatic tumors in which the color and gain of the images have been adjusted to better illustrate the focal uptake of the radiotracer in the liver. The black arrows on the images (right) denote areas of significant radiotracer uptake, signifying the presence of metastatic SCLC lesions.

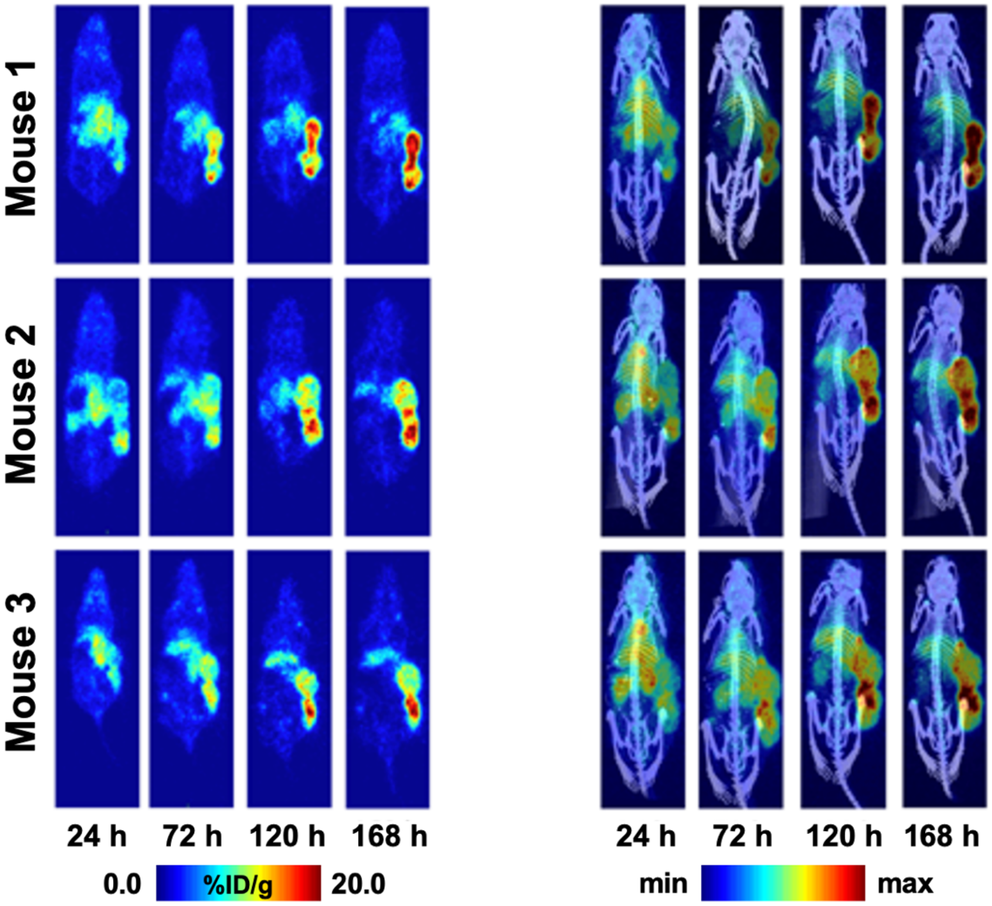

Figure S5. PET images of mice bearing subcutaneous PDX-1231 xenografts that had been injected with [^{89}Zr]Zr-^{degly}DFO-αCD133 [3.7–3.9 MBq (100–105 μCi), 5–5.5 μg in 100 μL of PBS] (n = 3). Coronal slices are shown on the left, while MIPs are shown on the right. These data correspond with that shown in **Table S3**.

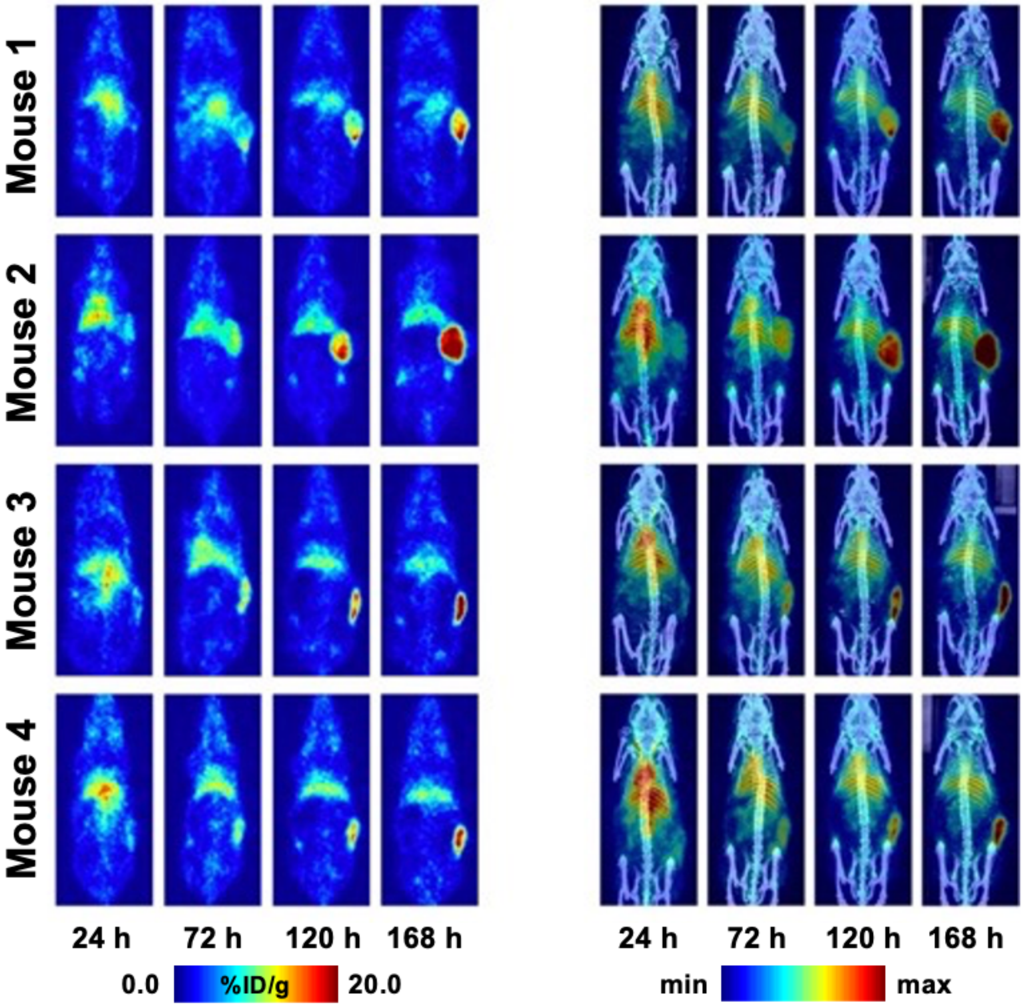

Figure S6. PET images of mice bearing subcutaneous PDX-599 xenografts that had been injected with [^{89}Zr]Zr-^{degly}DFO-αCD133 [3.7–3.9 MBq (100–105 μCi), 5–5.5 μg in 100 μL of PBS] (n = 3). Coronal slices are shown on the left, while MIPs are shown on the right. These data correspond with that shown in **Table S3**.

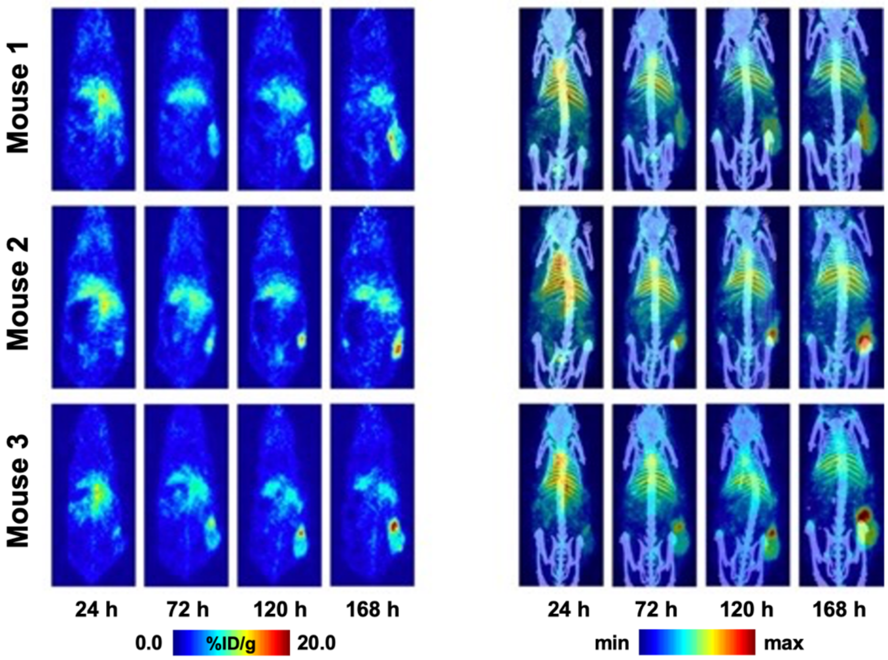

Figure S7. PET images of mice bearing subcutaneous PDX-973 xenografts that had been injected with [^{89}Zr]Zr-^{degly}DFO-αCD133 [3.7–3.9 MBq (100–105 µCi), 5–5.5 µg in 100 µL of PBS] (n = 3). Coronal slices are shown on the left, while MIPs are shown on the right. These data correspond with that shown in **Table S3**.

SUPPLEMENTAL TABLES

**Antibody**	**k_a (M^{-1} s^{-1})**	**k_d (s^{-1})**	**K_D (M)**
**αCD133**	2.85×10^5 ($\pm$ 9.26×10^1)	1.79×10^{-4} ($\pm$ 1.59×10^{-6})	6.92×10^{-10} ($\pm$ 6.39×10^{-12})
**DFO-αCD133**	7.78×10^4 ($\pm$ 3.93×10^1)	1.23×10^{-4} ($\pm$ 1.93×10^{-5})	1.58×10^{-9} ($\pm$ 2.49×10^{-10})

Table S1. Binding parameters for αCD133 and DFO-αCD133 with recombinant CD133 as measured via surface plasmon resonance. These data correspond with that shown in **Figure 1B**.

Tissue	Subcutaneous H82	Orthotopic H82-*luc*	Metastatic H82-*luc*
**Blood**	10.1 ± 1.9	6.9 ± 4.7	0.5 ± 0.7
**Tumor**	50.8 ± 7.7	34.9 ± 18.0	n/a
**Heart**	2.4 ± 1.0	1.7 ± 1.1	1.4 ± 0.1
**Lungs**	3.7 ± 1.7	3.7 ± 1.6	1.3 ± 0.6
**Liver**	4.8 ± 2.4	4.5 ± 1.6	31.8 ± 8.8
**Spleen**	4.5 ± 2.1	3.0 ± 1.1	16.7 ± 2.6
**Pancreas**	0.6 ± 0.1	0.4 ± 0.1	0.3 ± 0.0
**Stomach**	0.5 ± 0.2	0.2 ± 0.1	0.4 ± 0.1
**S. Intestine**	0.8 ± 0.5	0.3 ± 0.2	0.7 ± 0.1
**L. Intestine**	0.5 ± 0.1	0.6 ± 0.2	0.4 ± 0.1
**Kidney**	2.1 ± 1.3	2.4 ± 1.8	4.6 ± 1.3
**Muscle**	0.4 ± 0.2	0.2 ± 0.1	0.1 ± 0.1
**Femur**	4.3 ± 1.0	2.0 ± 0.5	13.6 ± 5.3
**Skin**	2.1 ± 0.6	1.2 ± 0.5	1.1 ± 0.3
**Tail**	1.3 ± 0.1	1.4 ± 1.1	1.4 ± 0.5

Table S2. Biodistribution data collected at 144 h (subcutaneous and orthotopic models) or 168 h (metastatic model) after the intravenous administration of [^{89}Zr]Zr-DFO-αCD133 (subcutaneous and orthotopic) or [^{89}Zr]Zr-^{degly}DFO- αCD133 (metastatic) [3.7–3.9 MBq (100–105 µCi), 5–5.5 µg in 100 µL of PBS] to athymic nude (subcutaneous and orthotopic) or NSG (metastatic) tumor-bearing mice. The values for the subcutaneous model were originally reported in 2022 by Kunihiro, *et al*..[2] Values are presented in units of %ID/g and are expressed as the mean ± standard deviation. These data correspond with that shown in **Figures S2-4**.

Tissue	PDX-1231	PDX-599	PDX-973
**Blood**	6.7 ± 2.9	9.0 ± 1.4	8.9 ± 1.2
**Tumor**	33.6 ± 7.6	21.4 ± 12.8	20.7 ± 11.9
**Heart**	3.1 ± 0.8	2.6 ± 0.3	3.3 ± 0.4
**Lungs**	3.5 ± 1.7	2.2 ± 0.4	2.5 ± 0.3
**Liver**	6.1 ± 0.4	3.6 ± 0.8	3.7 ± 0.9
**Spleen**	22.7 ± 12.1	10.0 ± 4.9	28.5 ± 9.1
**Pancreas**	0.6 ± 0.1	0.4 ± 0.3	0.5 ± 0.1
**Stomach**	0.6 ± 0.4	0.4 ± 0.1	0.4 ± 0.1
**S. Intestine**	0.6 ± 0.1	0.6 ± 0.2	0.6 ± 0.2
**L. Intestine**	0.7 ± 0.1	0.9 ± 0.6	0.4 ± 0.1
**Kidney**	6.3 ± 0.8	2.3 ± 0.5	1.8 ± 0.5
**Ovaries**	3.2 ± 0.7	2.7 ± 1.6	2.6 ± 1.2
**Muscle**	0.4 ± 0.2	0.6 ± 0.3	0.7 ± 0.6
**Femur**	6.7 ± 2.1	3.6 ± 1.2	4.6 ± 0.6
**Skin**	7.3 ± 1.7	4.8 ± 0.3	6.4 ± 1.4
**Tail**	1.4 ± 0.1	1.2 ± 0.2	1.2 ± 0.1

Table S3. Biodistribution data collected 168 h after the intravenous administration of [^{89}Zr]Zr-^{degly}DFO-αCD133 [3.7–3.9 MBq (100–105 μCi), 5–5.5 μg in 100 μL of PBS] to NSG tumor-bearing mice [PDX-1231 (n = 3), PDX-599 (n = 4), PDX-973 (n = 3)]. Values are in units of %ID/g and are expressed as mean ± standard deviation. This data corresponds with data shown in **Figures S5–S7**.

Organ/tissue	Absorbed dose coefficient [Gy/MBq]	Absorbed dose [Gy] for 4.6 MBq administration	Absorbed dose [Gy] for 9.2 MBq administration
**Heart**	0.793	3.65	7.30
**Lungs**	0.736	3.39	6.77
**Liver**	1.89	8.67	17.3
**Spleen**	0.678	3.12	6.24
**Pancreas**	0.679	3.12	6.25
**Stomach**	0.695	3.20	6.39
**S. Intestine**	0.687	3.16	6.32
**L. Intestine**	0.682	3.14	6.28
**Gallbladder**	0.852	3.92	7.83
**Kidneys**	0.687	3.16	6.32
**Red marrow**	0.685	3.15	6.31
**Urinary bladder**	0.671	3.09	6.17
**Brain**	0.684	3.14	6.29
**Thyroid**	0.725	3.34	6.67
**Tumor**	6.68	30.7	61.5
**Skin**	0.445	2.05	4.09
**Other tissues**	0.682	3.14	6.28

Table S4. Dosimetry values for [^{177}Lu]Lu-DTPA-A"-CHX-αCD133 estimated from [^{89}Zr]Zr-DFO-αCD133 PET imaging data acquired in mice bearing subcutaneous H82 tumors.[2] These data were used to inform the longitudinal radioimmunotherapy study with [^{177}Lu]Lu-DTPA-A"-CHX-αCD133.

Cohort	Mouse Number	Survival (# days)	Reason for Euthanasia
Saline	1	11	Necrotic Tumor
	2	8	Tumor >2000 mm^3
	3	33	Tumor >2000 mm^3
	4	26	Tumor >2000 mm^3
	5	29	Tumor >2000 mm^3
	6	11	Tumor >2000 mm^3
	7	33	Tumor >2000 mm^3
	8	29	Tumor >2000 mm^3
	9	26	Tumor >2000 mm^3
	10	26	Tumor >2000 mm^3
DTPA-A″-CHX-αCD133	11	33	Tumor >2000 mm^3
	12	19	Tumor >2000 mm^3
	13	19	Lost >10% of Body Weight
	14	8	Tumor >2000 mm^3
	15	26	Tumor >2000 mm^3
	16	11	Tumor >2000 mm^3
	17	26	Tumor >2000 mm^3
	18	8	Tumor >2000 mm^3
	19	26	Tumor >2000 mm^3
	20	26	Tumor >2000 mm^3
[^{177}Lu]Lu-DTPA-A″-CHX-αCD133 (4.6 MBq)	21	36	Tumor >2000 mm^3
	22	26	Tumor >2000 mm^3
	23	40	Tumor >2000 mm^3
	24	26	Tumor >2000 mm^3
	25	40	Tumor >2000 mm^3
	26	33	Tumor >2000 mm^3
	27	33	Tumor >2000 mm^3
	28	19	Tumor >2000 mm^3
	29	33	Tumor >2000 mm^3
	30	33	Tumor >2000 mm^3
[^{177}Lu]Lu-DTPA-A″-CHX-αCD133 (9.3 MBq)	31	68	Tumor >2000 mm^3
	32	63	Tumor >2000 mm^3
	33	83	Tumor >2000 mm^3
	34	63	Tumor >2000 mm^3
	35	49	Tumor >2000 mm^3
	36	83	Tumor >2000 mm^3
	37	83	Tumor >2000 mm^3
	38	77	Tumor >2000 mm^3
	39	26	Tumor >2000 mm^3
	40	43	Tumor >2000 mm^3

Table S5. Survival results for each of the mice in the longitudinal therapy study.

REFERENCES

1. Sharma, S. K.; Lyashchenko, S. K.; Park, H. A.; Pillarsetty, N.; Roux, Y.; Wu, J.; Poty, S.; Tully, K. M.; Poirier, J. T.; Lewis, J. S., A rapid bead-based radioligand binding assay for the determination of target-binding fraction and quality control of radiopharmaceuticals. *Nucl Med Biol* **2019,** *71*, 32-38.
2. Kunihiro, A. G.; Sarrett, S. M.; Lastwika, K. J.; Solan, J. L.; Pisarenko, T.; Keinänen, O.; Rodriguez, C.; Taverne, L. R.; Fitzpatrick, A. L.; Li, C. I.; Houghton, A. M.; Zeglis, B. M.; Lampe, P. D., CD133 as a Biomarker for an Autoantibody-to-ImmunoPET Paradigm for the Early Detection of Small Cell Lung Cancer. *J Nucl Med* **2022,** *63* (11), 1701-1707.
